# Potential therapeutic effects of apigenin for colorectal adenocarcinoma: A systematic review and meta‐analysis

**DOI:** 10.1002/cam4.70171

**Published:** 2024-09-10

**Authors:** Koohyar Ahmadzadeh, Shayan Roshdi Dizaji, Fatemeh Ramezani, Farnad Imani, Jebreil Shamseddin, Arash Sarveazad, Mahmoud Yousefifard

**Affiliations:** ^1^ Physiology Research Center Iran University of Medical Sciences Tehran Iran; ^2^ Pain Research Center, Department of Anesthesiology and Pain Medicine Iran University of Medical Sciences Tehran Iran; ^3^ Infectious and Tropical Diseases Research Center Hormozgan Health Institute, Hormozgan University of Medical Sciences Bandar Abbas Iran; ^4^ Colorectal Research Center Iran University of Medical Sciences Tehran Iran; ^5^ Nursing Care Research Center Iran University of Medical Sciences Tehran Iran; ^6^ Pediatric Chronic Kidney Disease Research Center Tehran University of Medical Sciences Tehran Iran

**Keywords:** Apigenin, cell viability, colorectal cancer, flavonoids, tumor size

## Abstract

**Purpose:**

Therapeutic management of colorectal cancer (CRC) does not yet yield promising long‐term results. Therefore, there is a need for further investigation of possible therapeutic options. Various experiments have studied the effects of apigenin on CRC and have shown conflicting results. This systematic review and meta‐analysis investigates the currently existing evidence on the effect of apigenin on CRC.

**Methods:**

Medline, Embase, Scopus, and Web of Science databases were searched for articles related to apigenin and its effect on CRC in the preclinical setting. Cell viability, growth inhibition, apoptosis, and cell cycle arrest for in‐vitro, and body weight, tumor size, and mortality in in‐vivo studies were extracted as outcomes.

**Results:**

Thirty‐nine articles investigating colorectal adenocarcinoma were included in this meta‐analysis. Thirty‐seven of these studies had data for in vitro experiments, with eight studies having data for in vivo experiments. Six articles had both in vitro and in vivo assessments. Our analysis showed apigenin reduces cell viability and induces growth inhibition, apoptosis, and cell cycle arrest in in vitro studies. The few in vivo studies indicate that apigenin decreases tumor size while showing no effects on the body weight of animal colorectal adenocarcinoma models.

**Conclusion:**

Our results demonstrated that apigenin, through reducing cell viability, inducing growth inhibition, apoptosis, and cell cycle arrest, and also by decreasing the tumor size, can be considered as a possible adjuvant agent in the management of colorectal adenocarcinoma. However, further in vivo studies are needed before any efforts to translate the current evidence into clinical studies.

## 1 INTRODUCTION

Colorectal cancer (CRC) is one of the most commonly diagnosed cancers, and with estimates of 1.9 million new cases and 1 million deaths in 2022, it is ranked third among cancers in incidence and second in mortality.^1^ The main therapies for CRC are surgery and chemotherapy. However, even with aggressive therapies, patients with CRC still have significant recurrence rates of as high as 50%.^2^ Moreover, the adverse effects of currently used medications and the minimal choice of effective drugs limit the treatment of CRC.^3^ This demonstrates an ongoing need for other alternatives, either as the main line of treatment or as adjuvants to currently existing therapies, such as lifestyle and dietary modifications.^4^

Diets rich in fruits and vegetables have been associated with a lower incidence of cancers such as CRC^5^ and recent studies indicate that extracts obtained from edible plants may have anti‐cancer properties.^6^ Flavonoids, a group of naturally occurring polyphenolic compounds widely present in plants, have been known to pose anti‐inflammatory, antioxidant, and also anticarcinogenic effects by modulating various cellular processes, such as glycolysis, apoptosis, and DNA repair.^7^, ^8^ Flavonoids are divided into six major classes—flavonols (e.g., quercetin), flavones (e.g., Apigenin), isoflavonoids (e.g., genistein), flavans, flavanones, and anthocyanins.^9^ Flavones have various substitution patterns giving them a wide range of biological activity with effects against a range of cancer cells, including breast, prostate, lung, and hematologic cancers.^7^, ^10^, ^11^, ^12^, ^13^

Apigenin is one of the well‐known flavonoids that exists in many vegetables and fruits and has been shown to have potential chemotherapeutic effects against neoplastic cells and limited toxic and no mutagenic effects on normal cells.^14^, ^15^ Studies have shown that apigenin affects cell growth, cell cycle, and apoptosis in different cancer cell lines.^15^, ^16^ These antineoplastic effects have been linked to the modifications caused on cellular pathways such as nuclear factor kappa B, protein kinase, and WNT/ β‐catenin and also through modulation of survival and death effectors such as STAT3, MCL‐1, and PI3K.^15^, ^17^

Studies on the effects of apigenin on CRC, including in vitro and in vivo research, have reported conflicting results. This systematic review and meta‐analysis investigates and strengthens the currently existing evidence on the effects of apigenin on CRC in preclinical studies.

## 2 METHOD

### 2.1 Study design and search strategy

The present systematic review and meta‐analysis was designed to assess the effectiveness of apigenin on CRC. PICO was defined as: Problem (P): CRC cell line or CRC animal model, Intervention (I): apigenin administration, Comparison (C): control group with no apigenin administration. Outcome (O): cell viability, apoptosis, growth inhibition, cell cycle arrest for in vitro studies and tumor size, body weight, and mortality for animal models.

Keywords were chosen based on MeSh terms (Medline database) and Emtree terms (Embase) with the help of experts in the field and also review of related literature. An extensive search was performed on four online databases (PubMed, Embase, Scopus, and Web of Science) until March 31st, 2024 (Data S1, search strategy). Google and Google Scholar search engines and the references of related articles were used to retrieve any possibly missed articles.

Selection Criteria:

All in vivo and in vitro studies, studying the effect of apigenin administration on CRC were included. Exclusion criteria were lack of a nontreated CRC control group, not reporting the desired outcomes, not reporting the required data, human studies, duplicate studies, and studies reporting combination apigenin therapy or derivates of apigenin.

### 2.2 Data extraction and risk of bias

Two reviewers independently performed title and abstract screening of retrieved records, and relevant articles were included after a full‐text review. Information provided by the studies was filled into a checklist, and any disputes were resolved by consulting a third reviewer. The extracted data were study characteristics (author name, publication year), cell or animal model used, sample size, type, dose, duration, and interval of apigenin administration, and studied outcome. Data presented in figures and charts were extracted using Plot Digitizer software (version 2.0; https://plotdigitizer.sourceforge.net.).

The risk of bias in in vitro studies was assessed by the guidelines provided by the National Toxicology Program^18^ and SYRCLE's risk of bias tool was used for the quality assessment of the in vivo studies.^19^ National Toxicology Program guidelines assess the risk of bias in domains of randomization, blinding, experimental conditions, outcome assessment and analysis, exposure characterization, and other potential biases. SYRCLE's tool assesses the risk of bias in domains of randomization, blinding, baseline characteristics, outcome assessment and reporting, incomplete data assessment, and other risks of bias.

### 2.3 Statistical analysis

All analyses were performed using STATA 17.0 statistical software. Gathered data were mean and standard deviation (SD), and overall results are reported as standardized mean difference (SMD) and 95% confidence interval (CI) using the meta package of the statistical software. Statistical analysis was performed in two sections of in vitro and in vivo experiments. Desired outcomes included cell viability, cell apoptosis, cell growth inhibition, and cell cycle arrest for in in vitro studies and tumor size, body weight, and mortality for in vivo studies.

Since the follow‐up duration varied among studies, the analysis was stratified by duration of follow‐up (24‐h, 48‐h, 72‐h). We also provided subgroup analysis for all outcomes according to the administrated doses.

Heterogeneity was assessed using *I* ^2^ statistics and the Chi‐squared test (*I* ^2^ greater than 50% demonstrates the presence of heterogeneity). Since considerable heterogeneity was expected among the included studies, a random effect model analysis was performed. Finally, publication bias was investigated by Egger's test and visualized by a funnel plot.

## 3 RESULTS

### 3.1 Study Characteristics

**FIGURE 1:**
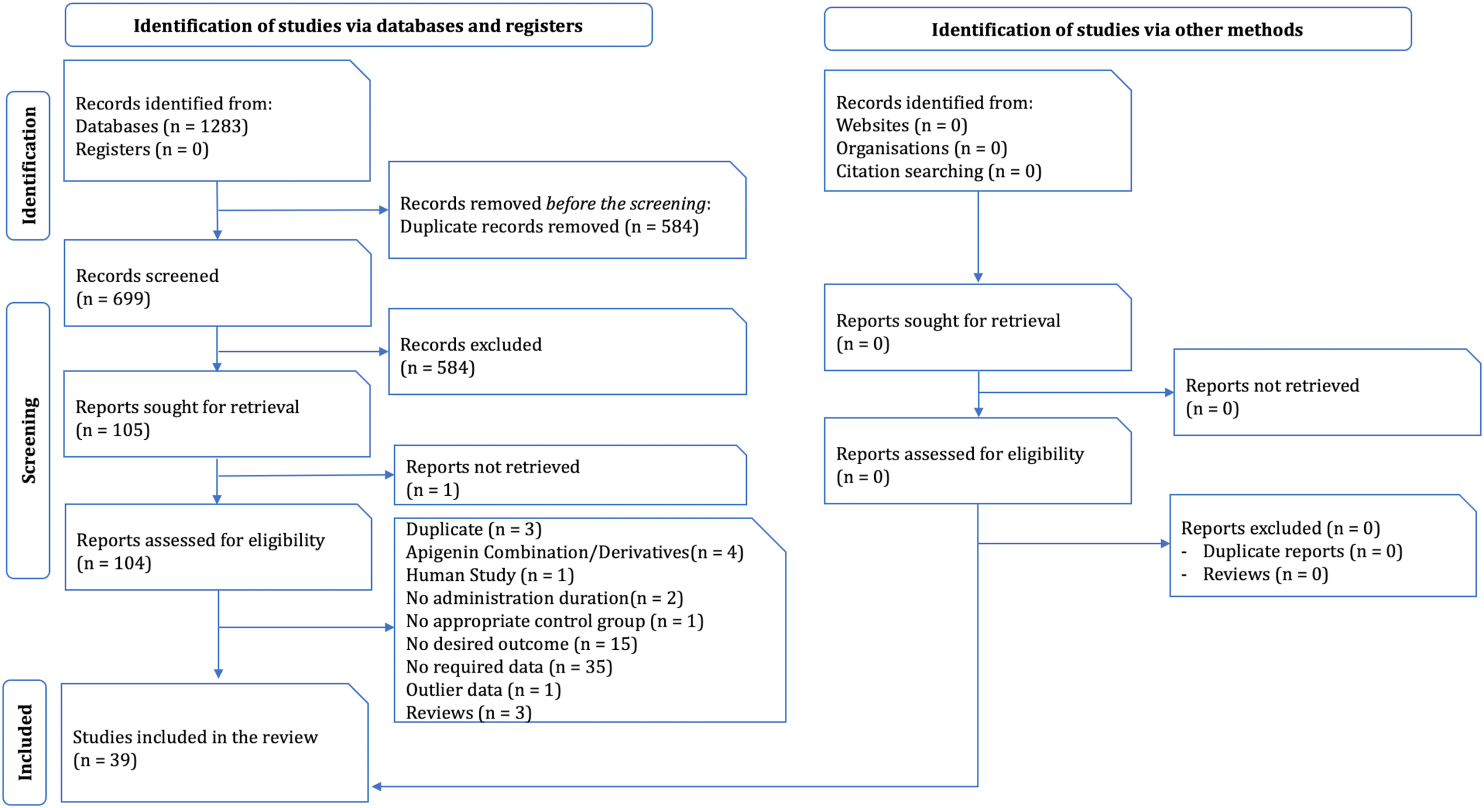
PRISMA flow diagram for study selection.

The systematic search resulted in 699 nonduplicate reports; from which 105 papers were found to be eligible. After further evaluation, 39 articles were included in this study (Figure 1).

Thirty‐seven articles were in‐vitro studies^7^, ^8^, ^13^, ^15^, ^16^, ^17^, ^18^, ^19^, ^20^, ^21^, ^22^, ^23^, ^24^, ^25^, ^26^, ^27^, ^28^, ^29^, ^30^, ^31^, ^32^, ^33^, ^34^, ^35^, ^36^, ^37^, ^38^, ^39^, ^40^, ^41^, ^42^, ^43^, ^44^, ^45^, ^46^, ^47^, ^48^ and eight articles were in‐vivo studies.^15^, ^18^, ^22^, ^35^, ^39^, ^48^, ^49^, ^50^ Six articles had both in vitro and in vivo assessments. All studies investigated colorectal adenocarcinoma cell lines and animal models. The studies included results for the administration of various amounts of apigenin, ranging from amounts less than 10 to 2777 μM for in vitro and 25 to 300 mg/kg for in vivo studies for follow‐ups of 24–72 h. Characteristics of included studies are reported in more detail in each of the respective proceeding sections and tables.

### 3.2 Effect of apigenin administration on colorectal adenocarcinoma cell lines

- **In Vitro Studies**

The 37 included in vitro studies reported outcomes of cell viability, growth inhibition, apoptosis, and cell cycle arrest. Effects of apigenin were studied on various cell lines of colorectal adenocarcinoma, with HCT‐116, HT‐29, and SW‐480 being the most common of them. Apigenin was administered with doses as low as 10^−4^ μM up to 2777 μM and follow‐up times of 24, 48, or 72 h. The most frequently administered dosages were between 5 to 200 μM. Table 1 demonstrates the characteristics of the included in vitro studies.

**TABLE 1:** In vitro studies characteristics.

Author, Year	Studied cell type(s)	Apigenin Administered Dose(s) (μM)	Apigenin Administration Duration(s) (hours)	Outcome(s)			
				Cell viability	Apoptosis	Growth inhibition	Cell Cycle
Banerjee, 2017^18^	HCT‐15, HT‐29	6.25, 12.5, 25, 50, 100, 200	48	✓			
Buhagiar, 2008^19^	HCT‐116	1, 10, 100	48	✓			
Cheng, 2021^20^	HCT‐116	1, 10, 20, 40, 80, 120, 160	24, 48	✓	✓		
Chidambara, 2012^7^	SW‐480	12.5, 25, 50, 100, 200	24, 48	✓			
Cho, 2015^21^	HT‐29	50, 100, 200	48	✓			
Chung, 2007^16^	HT‐29 APC, HT‐29 GaL	20, 30, 40, 60, 80	48				✓
Chunhua, 2013^22^	DLD‐1, LS174T, SW‐480	20, 40, 80, 120	24, 48	✓			
Cicek, 2023^23^	HT‐29	6.25, 12.5, 25	24, 48	✓			
Dai, 2016^24^	DLD‐1, SW‐480	10, 20, 40, 60	24, 48	✓			
Farah, 2003^25^	HCT‐116, HT‐29	7.5, 20, 40	24	✓			
Fernandez, 2021^8^	HCT‐116, HT‐29, T84	10, 20, 30, 40, 50	48	✓			
Hong, 2022^26^	HCT‐116, HT‐29	5, 10, 20	24, 72	✓			✓
Iizumi, 2013^27^	HT‐29	20, 40, 60, 80	24				✓
Kim, 2008^28^	SNU‐C4	1	24			✓	
Klampfer, 2004^29^	HCT‐116	5, 10, 20	24, 48		✓		
Lee, 2009^30^	SNU‐C4	0.0001, 0.001, 0.01, 0.1, 1, 10	72			✓	
Lee, 2014^31^	HCT‐116	6.25, 12.5, 25, 50	24	✓			
Richter, 1999^32^	SW‐480	1, 5, 10, 50, 100	48	✓	✓		
Sain, 2023^33^	COLO‐205	10, 20, 40, 60, 80, 120	24, 48	✓			
Shan, 2017^34^	DLD‐1, HCT‐116, HT‐29	10, 20, 40, 60	24	✓			
Shao, 2013^15^	DLD‐1, HCT‐8, HCT‐116, HT‐29, SW‐48	10, 15, 20, 30	24, 48, 72			✓	
Shi, 2023^35^	HCT‐8, LS‐174 T	40, 60, 80	24	✓	✓		
Simsek, 2013^36^	DLD‐1	370.3, 925.75, 1851.49, 2777.24	24, 48, 72	✓			
Smiljkovic, 2017^37^	HCT‐116	20, 40, 60, 80	48	✓			
Takagaki, 2005^38^	HT‐29	5, 10, 20, 30, 60	24				✓
Tong, 2019^39^	HCT‐116, LOVO	1.56, 3.13, 6.25, 12.5, 25, 50, 100, 200, 400	24	✓			
Turktekin, 2011^40^	HT‐29	15, 45, 75, 90, 100	24, 48, 72	✓			
Wang, 2000^41^	Caco2, HT‐29, SW‐480	10, 20, 30, 40, 50, 60, 70, 80	24, 48				✓
Wang, 2004^42^	Caco2, SW‐480	20, 40, 60, 80	48				✓
Wang, 2013^13^	SW‐480	5, 10, 20, 40, 60, 80, 100	24	✓			
Wang, 2016^43^	HCT‐116	60	24, 48, 72			✓	
Wang. B, 2017^44^	HCT‐116	20, 40, 80, 120, 160	24, 48, 72			✓	
Wang. J, 2017^45^	SW‐620	10, 20, 40, 80	24, 48, 72	✓	✓		
Xu, 2016^17^	HCT‐15, SW‐480	5, 10, 20, 40, 80	48	✓			
Yang, 2021^46^	HCT‐116, HT‐29, HCT‐116‐5‐FUR^a^	20	48	✓	✓		✓
Zhang, 2021^47^	HCT‐116, SW‐480	5, 25, 50, 100	24, 48, 72	✓	✓		
Zhong, 2010^48^	HCT‐116	1, 10	72		✓		

^a^ 5‐Fluorouracil resistant.

### 3.3 Cell Viability

Results of the effect of apigenin on cell viability show that compared to untreated cells, apigenin reduces cell viability during follow‐up time of 24 h (overall SMD = −5.51, 95% CI: −6.43 to −4.59, *p* < 0.0001; *I* ^2^:97.22%, *p* < 0.0001), 48 h (SMD = −6.92, 95% CI: −8.14 to −5.70, *p* < 0.0001; *I* ^2^:97.14%, *p* < 0.0001) and 72 h (SMD = −12.99, 95% CI: −16.39 to −9.60, *p* < 0.0001; *I* ^2^:96.65%, *p* < 0.0001). Subgroup analysis showed that apigenin significantly reduces cell viability in all administrated doses except in doses between 200 to 1000 μM at 24 h (*p* = 0.052) and between 40–50 μM (*p* = 0.057) and 70–80 μM (*p* = 0.145) at 72 h. More detailed data on the effects of various administration doses on cell viability in each time period is demonstrated in Table 2 and Figures S1–S13.

**TABLE 2:** Effect of different doses of apigenin on colorectal adenocarcinoma cell line viability.

Subgroups	*N*	SMD (95% CI)	*P*	*I* ^2^% (P for heterogeneity)
24 h				
≤10	23	−2.22 (−3.11, −1.32)	<0.0001	98.6 (<0.0001)
10.1–20	19	−2.99 (−4.17, −1.81)	<0.0001	93.14 (<0.0001)
20.1–30	7	−15.29 (−23.67, −6.90)	<0.0001	91.41 (<0.0001)
30.1–40	13	−4.04 (−5.96, 02.14)	<0.0001	92.33 (<0.0001)
40.1–50	7	−13.55 (−20.82, −6.28)	<0.0001	96.25 (<0.0001)
50.1–60	8	−3.06 (−3.96, −2.17)	<0.0001	89.23 (0.001)
70.1–80	10	−4.48 (−5.77, −3.18)	<0.0001	93.24 (<0.0001)
90.1–100	8	−14.49 (−21.34, −7.64)	<0.0001	97.27 (<0.0001)
120 & 160	6	−4.68 (−6.44, −2.92)	<0.0001	0.00 (0.062)
200	3	−28.32 (−38.36, −18.27)	<0.0001	92.8 (<0.0001)
200.1–1000	4	−12.42 (−24.96, 0.12)	0.052	0.00 (0.323)
1000.1–2777	3	−13.74 (−18.37, −9.12)	<0.0001	40.22 (0.18)
Overall	111	−5.51 (−6.43, −4.59)	<0.0001	97.22 (<0.0001)
48 h				
≤10	22	−3.42 (−6.24, −0.61)	0.0017	98.42 (<0.0001)
10.1–20	22	−4.17 (−5.72, −2.61)	<0.0001	93.19 (<0.0001)
20.1–30	8	−14.27 (−21.16, −7.38)	<0.0001	89.32 (<0.0001)
30.1–40	14	−5.40 (−7.53, −3.27)	<0.0001	92.33 (<0.0001)
40.1–50	10	−10.22 (−15.18, −5.26)	<0.0001	95.5 (<0.0001)
50.1–60	5	−4.12 (−7.43, −0.81)	0.015	89.23 (0.001)
70.1–80	11	−7.72 (−10.77, 4.68)	<0.0001	93.24 (<0.0001)
90.1–100	11	−20.14 (−28.84, −11.44)	<0.0001	96.53 (<0.0001)
120 & 160	6	−5.39 (−6.64, −4.13)	<0.0001	0.00 (0.062)
200	4	−34.97 (−65.41, −4.52)	0.024	90.26 (<0.0001)
200.1–1000	2	−2.46 (−3.83, −1.10)	<0.0001	0.00 (0.323)
1000.1–2777	3	−10.84 (−15.81, −5.87)	<0.0001	40.22 (0.18)
Overall	118	−6.92 (−8.14, −5.70)	<0.0001	97.14 (<0.0001)
72 h				
≤10	7	−9.57 (−14.88, −4.27)	<0.0001	92.01 (<0.0001)
10.1–20	6	−14.09 (−21.57, −6.61)	<0.0001	90.62 (<0.0001)
20.1–30	2	−21.22 (−29.84, −12.60)	<0.0001	0.00 (0.5)
30.1–40	1	−27.25 (−38.20, −16.30)	<0.0001	NA
40.1–50	3	−19.50 (−39.58, 0.59)	0.057	88.43 (<0.0001)
70.1–80	2	−18.78 (−44.05, 6.50)	0.145	93.19 (<0.0001)
90.1–100	4	−20.99 (−37.77, −4.21)	0.014	97.18 (0.003)
200.1–1000	2	−2.00 (−3.23, −0.78)	0.001	0.00 (0.442)
1000.1–2777	3	−8.49 (−13.75, −3.24)	0.002	66.28 (0.054)
Overall	30	−12.99 (−16.39, −9.60)	<0.0001	96.65 (<0.0001)

*Note*: Doses presented as μM. Dose categories not present in the subgroup column did not have any reported data in the included studies.

Abbreviations: CI, confidence interval; SMD, standardized mean difference.

### 3.4 Growth Inhibition

Apigenin significantly induces growth inhibition compared to untreated cells in any of the follow‐up times of 24 h (SMD = 9.78, 95% CI: 4.19–15.38, *p* = 0.001; *I* ^2^:98.12%, *p* < 0.0001), 48 h (SMD = 11.11, 95% CI: 5.10–17.12, *p* < 0.0001; *I* ^2^:97.68%, *p* < 0.0001) and 72 h (SMD = 9.43, 95% CI: 4.83–14.03, *p* < 0.0001; *I* ^2^: 97.96%, *p* < 0.0001). Subgroup analysis showed that apigenin significantly induces growth inhibition in all administrated doses except in doses between 20 to 40 μM at 24 h (*p* = 0.183) and less than 20 μM at 48‐h (*p* = 0.059). The administered dosages were between 1 and 160 μM. More detailed results of each administered dose in each time period are shown in Table 3 and Figures S14–S17.

**TABLE 3:** Effect of different doses of apigenin on colorectal adenocarcinoma cell line growth inhibition.

Subgroups	*N*	SMD (95% CI)	*p*	*I* ^2^ (P for heterogeneity)
24 h				
≤20	6	2.08 (0.56, 3.59)	0.007	75.55 (<0.0001)
20.1–40	2	14.21 (−6.71, 35.13)	0.183	86.99 (0.006)
60.1–80	2	14.12 (8.33, 19.92)	<0.0001	0.00 (0.44)
120 & 160	2	30.04 (17.96, 42.11)	<0.0001	0.00 (0.757)
Overall	12	9.78 (4.19, 15.38)	0.001	98.12 (<0.0001)
48 h				
≤20	7	4.92 (−0.19, 10.03)	0.059	97.01 (<0.0001)
20.1–40	1	16.40 (7.03, 25.76)	0.001	NA
60.1–80	2	17.32 (6.60, 28.04)	0.002	49.27 (0.16)
120 & 160	2	27.57 (16.40, 38.73)	<0.0001	0.00 (0.512)
Overall	12	11.11 (5.10, 17.12)	<0.0001	97.68 (<0.0001)
72 h				
≤20	7	4.35 (1.29, 7.40)	0.005	95.87 (0.00)
20.1–40	1	17.71 (7.61, 27.82)	0.001	NA
60.1–80	2	16.21 (2.73, 29.69)	0.018	69.05 (0.072)
120 & 160	2	21.80 (13.03, 30.57)	<0.0001	0.00 (0.908)
Overall	12	9.43 (4.83, 14.03)	<0.0001	97.96 (<0.0001)

*Note*: Doses presented as μM. Dose categories not present in the subgroup column did not have any reported data in the included studies.

Abbreviations: CI, confidence interval; SMD, standardized mean difference.

### 3.5 Apoptosis

The results show that apigenin significantly increases apoptosis compared to untreated cells when administered for 24 h (SMD = 4.13, 95% CI: 0.86–7.40, *p* = 0.013; *I* ^2^ = 95.69%, *p* < 0.0001) and 48 h (SMD = 7.51, 95% CI: 2.59–12.44, *p* = 0.003; *I* ^2^: 98.63%, *p* < 0.0001). No significant effect was observed for results of 72 h (SMD:1.82, 95% CI: −0.63 to 4.27, *p* = 0.145, *I* ^2^:72.06, *p* = 0.058). It should be mentioned that for the time of 72 h only one study with two separate experiments was included. Therefore, results should be interpreted with caution. Subgroup analysis showed that apigenin had a significant effect on apoptosis only when administered as dosages between 10 and 100 μM for 24 h (SMD = 5.84, 95% CI: 1.93–9.75, *p* = 0.003; *I* ^2^:93.31%, *p* < 0.0001) and 48 h (SMD = 7.51, 95% CI: 2.59–12.44, *p* = 0.003; *I* ^2^:98.63%, *p* < 0.0001); whereas no significant effect was observed for doses less than 10 μM (Table 4 and Figures S18,S19).

**TABLE 4:** Effects of different doses of apigenin on colorectal adenocarcinoma cell line apoptosis.

Subgroups	*N*	SMD (95% CI)	*p*	*I* ^2^% (P for heterogeneity)
24 h				
≤10	2	−0.07 (−0.98, 0.84)	0.872	0.00 (0.651)
10.1–100	6	5.84 (1.93, 9.75)	0.003	93.31 (<0.0001)
Overall	8	4.13 (0.86, 7.40)	0.013	95.68 (<0.0001)
48 h				
≤10	3	3.87 (−3.43, 11.18)	0.299	97.97 (<0.0001)
10.1–100	10	8.92 (2.59, 15.26)	0.006	98.68 (<0.0001)
Overall	13	7.51 (2.59, 12.44)	0.003	98.63 (<0.0001)
72 h				
≤10	2	1.82 (−0.63, 4.27)	0.15	72.06 (0.058)

*Note*: Doses presented as μM. Dose categories not present in the subgroup column did not have any reported data in the included studies.

Abbreviations: CI, confidence interval; SMD, standardized mean difference.

### 3.6 Cell Cycle Arrest

Cell cycle arrest was reported as the percentage of cells in different cell cycles including SubG1, G0‐G1, G1, S, and G2‐M, after administration of 5 to 80 μM apigenin for 24 or 48 h compared to untreated cells (Table 5).

**TABLE 5:** Effect of different doses of apigenin on cell cycles in colorectal adenocarcinoma cell lines.

Subgroups	*N*	SMD (95% CI)	*p*	*I* ^2^% (P for heterogeneity)
G1				
24 h				
≤10	5	−0.47 (−1.09, 0.14)	0.13	0.00 (0.23)
10.1–40	13	−2.52 (−3.44, −1.60)	<0.0001	69.85 (<0.0001)
40.1–60	8	−4.49 (−6.77, −2.21)	<0.0001	66.84 (0.01)
60.1–80	4	−5.41 (−6.71, −4.11)	<0.0001	0.00 (0.41)
Overall	30	−3.02 (−3.83, −2.21)	<0.0001	78.84 (<0.0001)
48 h				
≤10	3	−1.14 (−2.31, 0.03)	0.06	44.54 (0.17)
10.1–40	13	−2.85 (−3.97, −1.73)	<0.0001	77.89 (<0.0001)
40.1–60	2	−4.05 (−6.14, −1.96)	<0.0001	70.42 (0.02)
60.1–80	2	−5.17 (−7.43, −2.90)	<0.0001	75.90 (0.00)
Overall	20	−3.29 (−4.14, −2.44)	<0.0001	78.78 (0.00)
SubG1				
24 h				
≤10	4	1.91 (1.08, 2.75)	<0.0001	42.78 (0.06)
10.1–40	4	3.67 (2.41, 4.93)	<0.0001	0.00 (0.39)
Overall	8	2.46 (1.63, 3.29)	<0.0001	51.65 (0.01)
G0‐G1				
24 h				
≤10	4	−1.88 (−2.49, −1.28)	<0.0001	0.00 (0.65)
10.1–40	4	−2.73 (−3.81, −1.65)	<0.0001	8.25 (0.46)
Overall	8	−2.10 (−2.62, −1.57)	<0.0001	0.00 (0.57)
48 h				
10.1–40	5	−0.44 (−1.31, 0.44)	0.33	65.36 (0.01)
40.1–60	1	−4.79 (−6.93, −2.66)	<0.0001	0.00 (0.57)
60.1–80	1	−6.06 (−8.65, −3.47)	<0.0001	0.00 (0.91)
Overall	7	−1.79 (−3.19, −0.40)	0.01	86.42 (<0.0001)
S				
24 h				
≤10	9	−0.08 (−0.54, 0.37)	0.72	31.32 (0.11)
10.1–40	17	−0.63 (−1.35, 0.09)	0.09	76.33 (<0.0001)
40.1–60	8	−2.55 (−5.67, 0.57)	0.11	92.04 (<0.0001)
60.1–80	4	−2.93 (−7.15, 1.29)	0.17	96.87 (<0.0001)
Overall	38	−0.54 (−1.01, −0.07)	0.02	74.32 (<0.0001)
48 h				
≤10	3	0.17 (−0.59, 0.93)	0.66	0.00 (0.42)
10.1–40	18	0.34 (0.07, 0.62)	0.01	0.00 (0.25)
40.1–60	3	0.30 (−0.43, 1.04)	0.42	50.89 (0.06)
60.1–80	3	0.34 (−0.49, 1.18)	0.42	69.45 (<0.0001)
Overall	27	0.34 (0.09, 0.58)	<0.01	29.27 (0.01)
G2‐M				
24 h				
≤10	9	0.26 (−0.17, 0.69)	0.24	21.04 (0.14)
10.1–40	17	2.35 (1.26, 3.44)	<0.0001	83.19 (<0.0001)
40.1–60	8	9.43 (4.97, 13.89)	<0.0001	69.04 (<0.0001)
60.1–80	4	10.98 (5.63, 16.33)	<0.0001	87.73 (<0.0001)
Overall	38	2.79 (1.81, 3.78)	<0.0001	90.81 (<0.0001)
48 h				
≤10	3	2.24 (−0.44, 4.92)	0.10	83.49 (0.03)
10.1–40	18	3.51 (2.16, 4.86)	<0.0001	88.17 (<0.0001)
40.1–60	3	9.09 (6.11, 12.07)	<0.0001	55.57 (0.03)
60.1–80	5	10.32 (8.03, 12.61)	<0.0001	35.79 (0.01)
Overall	29	6.45 (4.90, 8.01)	<0.0001	93.07 (<0.0001)

*Note*: Doses presented as μM.

Abbreviations: CI, confidence interval; SMD, standardized mean difference.

Apigenin significantly increases the percentage of cells in subG1 during first 24 h (overall SMD = 2.46, 95% CI: 1.63–3.29, *p* < 0.0001; *I* ^2^:51.65%, *p* = 0.012). Both dosages of less than 10 μM and 10 to 40 μM significantly increased the percentage of cells in subG1. There was no data in the time of 48‐h. In S phase, a decrease was observed in the percentage of cells in 24 h (SMD = −0.54, 95% CI: −1.01 to −0.07, *p* = 0.024; *I* ^2^:74.32%, *p* < 0.0001), but a significant increase was observed after 48 h (overall SMD = 0.34, 95% CI: 0.09–0.58, *p* = 0.007; *I* ^2^:29.27%, *p* = 0.008). Subgroup analysis showed that only the dosage of 10 to 40 μM of apigenin had a significant effect on the percentage of cells in S at 48‐h (*p* = 0.014).

Apigenin significantly increases the percentage of cells in G2‐M after 24 h (overall SMD = 2.79, 95% CI: 1.81–3.78, *p* < 0.0001; *I* ^2^:90.81%, *p* < 0.0001) and 48 h (SMD = 6.45, 95% CI: 4.90–8.01, *p* < 0.0001; *I* ^2^:93.07%, *p* < 0.0001). Subgroup analysis showed that only the doses of less than 10 μM of apigenin did not have a significant effect on the percentage of cells in G2‐M (*p*_for 24‐h_ = 0.24; *p*_for 48‐h_ = 0.10).

Apigenin significantly decreases the percentage of cells in G0‐G1 during first 24 h (overall SMD = −2.10, 95% CI: −2.62 to −1.57, *p* < 0.0001; *I* ^2^:0.00%, *p* = 0.571) and 48 h (SMD = −1.79, 95% CI: −3.19 to −0.40, *p* = 0.011; *I* ^2^:86.42%, *p* < 0.0001) and also decreases the percentage of cells in G1 during first 24 h (overall SMD = −3.02, 95% CI: −3.83 to −2.21, *p* < 0.0001; *I* ^2^:78.84%, *p* < 0.0001) and 48 h (SMD = −3.29, 95% CI: −4.14 to −2.44, *p* < 0.0001; *I* ^2^:78.78%, *p* < 0.0001). Subgroup analysis showed that the only dosage of less than 10 μM of apigenin did not have a significant effect on the percentage of cells in G1 (*p*_for 24‐h_ = 0.133; *p*_for 48‐h_ = 0.056).

### 3.7 In Vivo Studies

#### 3.7.1 Study characteristics

Eight articles had data for the effects of apigenin in vivo CRC experiments. These articles reported outcomes of tumor size and body weight in colorectal adenocarcinoma mouse models. Apigenin was administered as doses of 25–300 mg/kg. Experiments were performed on various mice species, including Balb/C, Athymic nude, Min, and C.B.‐17 SCID. In noncancerous species, azoxymethane (AOM), HT‐29, HCT‐8, HCT‐116, or SW‐480 cells were used for cancer induction in the experiment animal (Table 6).

**TABLE 6:** In vivo studies characteristics.

Author, Year	Species	Mean age^a^	Cancer induction method	Apigenin Administration	Followup (days)	Administration route	Outcome(s)		Sample Size (case/control)
							Body weight	Tumor size	
Ai, 2017^49^	Balb/C mice	8	AOM	200, 300 mg/kg daily	21	Oral	✓		10
Banerjee, 2017^18^	Athymic nude mice	4–6	HT‐29 cells	50 mg/kg First 5 days: 3 times, then biweekly	33	IV		✓	3
Bian, 2020^50^	BALB/C mice	4	AOM	30 mg/kg daily	28	Oral		✓	8
Chunhua, 2013^22^	BALB/C‐nu mice	6–8	SW480 cells	50 mg/kg NR	42	NR		✓	3
Shi, 2023^35^	Nu/nu mice	6–8	HCT‐8	25 mg/kg Every other day	35	Intraperitoneal	✓	✓	10
Shao, 2013^15^	C.B.‐17 SCID mice	7	HCT‐116 cells	25 mg/kg daily	21	Oral	✓	✓	5
Tong, 2019^39^	BALB/c nu/nu mice	NR	HCT‐116 cells	200, 300 mg/kg NR	14–15	Intragastric	✓	✓	3
Zhong, 2010^48^	APC Min/+ mice	NR	NA	25 mg/kg 50 mg/kg Every other day	30	Oral		✓	6/6 5/6

^a^ Reported as weeks.

Abbreviations: AOM, Azoxymethane; IV, Intravenous; NA, Not Applicable; NR, Not Reported.

#### 3.7.2 Tumor size

**FIGURE 2:**
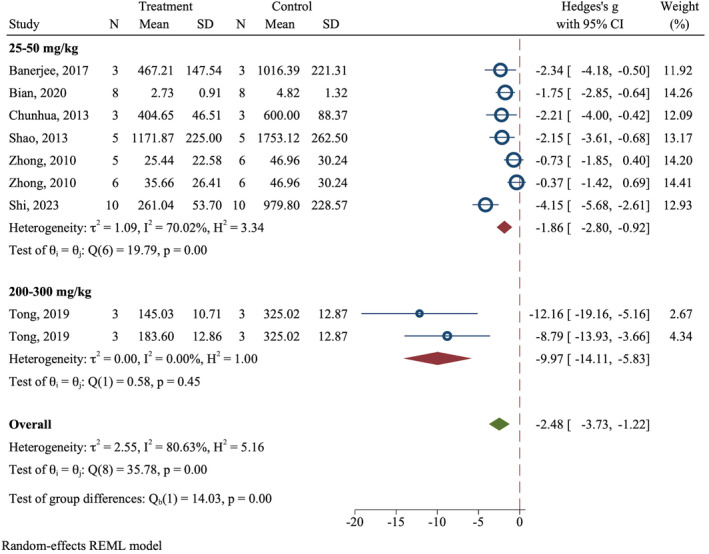
Effect of apigenin on tumor size of CRC animal models on the last day of follow‐up.

Meta‐analysis showed that apigenin can decrease the tumor size (Overall SMD = −2.48; 95% CI: −3.73 to −1.22, *p* = 0.003; *I* ^2^ = 80.63%; *p* < 0.0001) (Figure 2).

#### 3.7.3 Body weight

**FIGURE 3:**
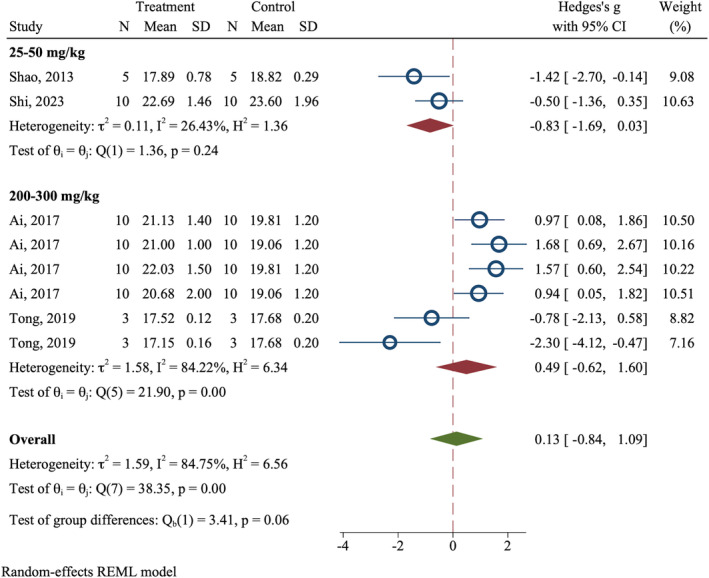
Effect of apigenin on CRC animal models body weight on the last day of follow‐up.

Meta‐analysis showed that the apigenin did not have a significant effect on animals' body weight (Overall SMD = 0.13; 95% CI: −0.84 to 1.09, *p* = 0.795; *I* ^2^ = 84.75%; *p* < 0.0001) (Figure 3).

#### 3.7.4 Risk of Bias and Publication bias

The quality of in vitro studies was assessed using the guidelines provided by the National Toxicology Program (Table 7). Overall, the risk of bias in domains of randomization and blinding could not be assessed due to no reported information in any of the studies and thus were rated as high in risk of bias, and in the remaining domains, the risk of bias was assessed to be low or very low in almost all studies.

**TABLE 7:** Risk of bias assessment for in vitro studies.

Study	Dose/Exposure level randomization	Group allocation concealment	Group experimental conditions	Personnel blinding	Outcome data analysis	Exposure characterization	Outcome assessment	Measured outcomes	Other potential biases
Banerjee, 2017^18^	High	High	Very low	High	Very low	Low	Low	Very low	Low
Buhagiar, 2008^19^	High	High	Very low	High	Very low	Low	Low	Very low	Low
Cheng, 2021^20^	High	High	Very low	High	Very low	Low	Low	Very low	Low
Chidambara, 2012^7^	High	High	Very low	High	Very low	Low	Low	Very low	Low
Cho, 2015^21^	High	High	Very low	High	Very low	Low	Low	Very low	Low
Chung, 2007^16^	High	High	Very low	High	Very low	Low	Low	Very low	Low
Chunhua, 2013^22^	High	High	Very low	High	Very low	Low	Low	Very low	Low
Cicek, 2023^23^	High	High	Very low	High	Very low	Low	Low	Very low	Low
Dai, 2016^24^	High	High	Very low	High	Very low	Low	Low	Very low	Low
Farah, 2003^25^	High	High	Very low	High	Very low	Low	Low	Very low	Low
Fernandez, 2021^8^	High	High	Very low	High	Very low	Low	Low	Very low	Low
Hong, 2022^26^	High	High	Very low	High	Very low	Low	Low	Very low	Low
Iizumi, 2013^27^	High	High	Very low	High	Very low	Low	Low	Very low	Low
Kim, 2008^28^	High	High	Very low	High	Very low	Low	Low	Very low	Low
Klampfer, 2004^29^	High	High	Very low	High	Very low	Low	Low	Very low	Low
Lee, 2009^30^	High	High	Very low	High	Very low	Low	Low	Very low	Low
Lee, 2014^31^	High	High	Very low	High	Very low	Low	Low	Very low	Low
Richter, 1999^32^	High	High	Very low	High	Very low	Low	Low	Very low	Low
Sain, 2023^33^	High	High	Very low	High	Very low	Low	Low	Very low	Low
Shan, 2017^34^	High	High	Very low	High	Very low	Low	Low	Very low	Low
Shao, 2013^15^	High	High	Very low	High	Very low	Low	Low	Very low	Low
Shi, 2023^35^	High	High	Very low	High	Very low	Low	Low	Very low	Low
Simsek, 2013^36^	High	High	Very low	High	Very low	Low	Low	Very low	Low
Smiljkovic, 2017^37^	High	High	Very low	High	Very low	Low	Low	Very low	Low
Takagaki, 2005^38^	High	High	Very low	High	Very low	Low	Low	Very low	Low
Tong, 2019^39^	High	High	Very low	High	Very low	Low	Low	Very low	Low
Turktekin, 2011^40^	High	High	Very low	High	Very low	Low	Low	Very low	Low
Wang, 2000^41^	High	High	Very low	High	Very low	Low	Low	Very low	Low
Wang, 2004^42^	High	High	Very low	High	Very low	Low	Low	Very low	Low
Wang, 2013^13^	High	High	Very low	High	Very low	Low	Low	Very low	Low
Wang, 2016^43^	High	High	Very low	High	Very low	Low	Low	Very low	Low
Wang. B, 2017^44^	High	High	Very low	High	Very low	Very low	Low	Very low	Low
Wang. J, 2017^45^	High	High	Very low	High	Very low	Low	Low	Very low	Low
Xu, 2016^17^	High	High	Very low	High	Very low	Low	Low	Very low	Low
Yang, 2021^46^	High	High	High	High	Very low	Low	Low	Very low	Low
Zhang, 2021^47^	High	High	Very low	High	Very low	Low	Low	Very low	Low
Zhong, 2010^48^	High	High	Very low	High	Very low	Low	Low	Very low	Low

The quality of in vivo studies was assessed using SYRCLE's risk of bias assessment tool. Based on our judgments, it was shown that the only domain with a low risk of bias was baseline characteristics. Studies were rated in the domain of outcome assessor blinding as low in one and unclear in the rest of the studies. Studies were evaluated as unclear in incomplete data assessment, randomization, and other domains of blinding (Table 8).

**TABLE 8:** Risk of bias assessment for in vivo studies.

Study	Allocation sequence	Baseline characteristics	Allocation concealment	Random housing	Investigator blinding	Outcome assessment random selection	Outcome assessor blinding	Incomplete data assessment	Selective outcome reporting	Other risks of bias
Ai, 2017^49^	Unclear	Low	Unclear	Unclear	Unclear	Unclear	Low	Unclear	Low	Low
Banerjee, 2017^18^	Unclear	Low	Unclear	Unclear	Unclear	Unclear	Unclear	Unclear	Unclear	Low
Bian, 2020^50^	Unclear	Low	Unclear	Unclear	Unclear	Unclear	Unclear	Unclear	Unclear	Low
Chunhua, 2013^22^	Unclear	Low	Unclear	Unclear	Unclear	Unclear	Unclear	Unclear	Unclear	Low
Shi, 2023^35^	Unclear	Low	Unclear	Unclear	Unclear	Unclear	Unclear	Unclear	Unclear	Low
Shao, 2013^15^	Unclear	Low	Unclear	Unclear	Unclear	Unclear	Unclear	Unclear	Unclear	Low
Tong, 2019^39^	Unclear	Low	Unclear	Unclear	Unclear	Unclear	Unclear	Unclear	Unclear	Low
Zhong, 2010^48^	Unclear	Low	Unclear	Unclear	Unclear	Unclear	Unclear	Unclear	Unclear	Low

**FIGURE 4:**
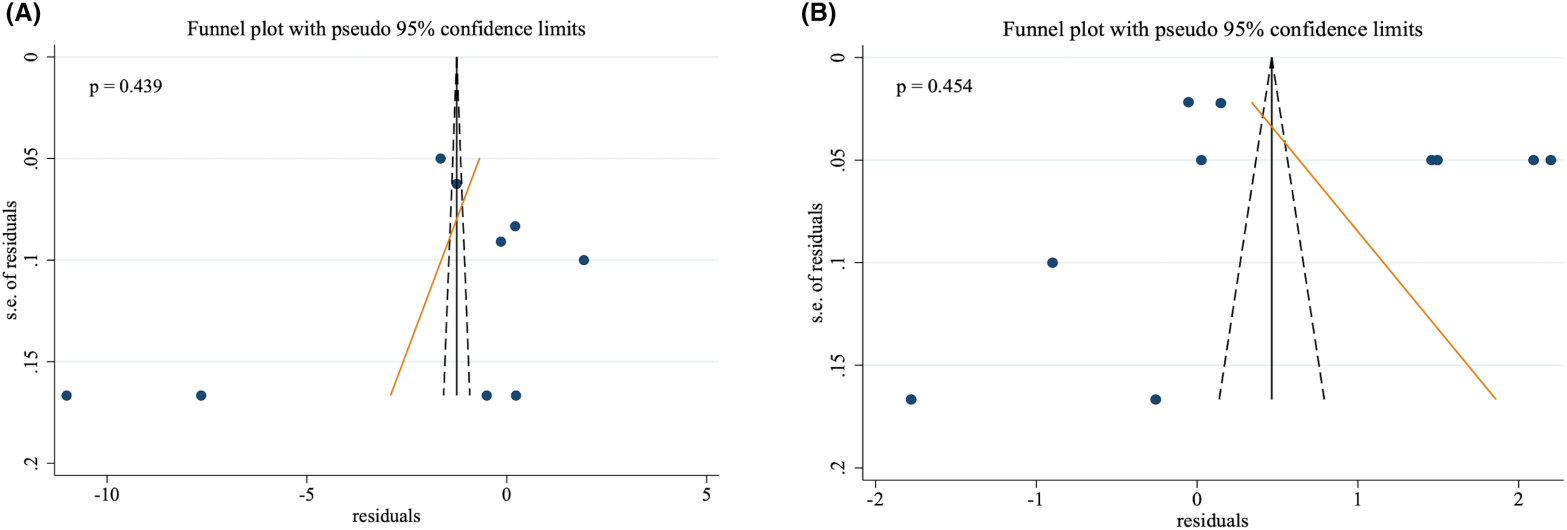
Publication bias of animal model studies.

Egger's test was used for publication bias assessment in in vivo studies. The results indicated no publication bias between in vivo studies in either of the outcomes (*p* tumor size = 0.439, *p* body weight = 0.454, Figure 4).

## 4 DISCUSSION

Our analysis for the in vitro studies showed that apigenin reduces cell viability and induces growth inhibition and apoptosis in colorectal adenocarcinoma cell lines. Apigenin was also shown to cause cell cycle arrest. Our results indicate that, in line with most studies, apigenin increases the percentage of cells in Subg1, G2‐M, and S while reducing the percentage of cells in the G0‐G1 and G1 phases. The only difference with most of the current evidence is the observed effect of an increase in the percentage of S cycle cells, which should be evaluated with caution, considering that in the subgroup analysis, most administered doses of apigenin did not show an effect of increasing the percentage of cells in the S cycle.

Our study results indicate that apigenin induces early apoptosis in colorectal adenocarcinoma cells, although this observed effect is limited by the scarce number of experiments performed on this outcome. Apigenin has a polypharmacological role in promoting apoptosis both by intrinsic and extrinsic pathways. Increasing the ratio of pro‐apoptotic to prosurvival markers (Bax/Bcl‐2), upregulation of antitumor p53, interruption of redux balance leading to ROS accumulation and intracellular Ca2+ dysregulation, and upregulation of death receptors and downstream caspase cascades are among apigenin‐induced apoptosis pathways.^43^, ^46^, ^47^ Apigenin also disturbs the cellular metabolism of neoplastic cells. Blockage of glycolysis by pyruvate kinase inhibition and enhanced catabolism of polyamines (i.e., a ROS scavenger) are some examples of apigenin's cellular metabolism disruption pathways.^34^, ^45^

The results of our study demonstrate that apigenin blockades the cell cycle at G0/G1 and G2/M checkpoints. Recently, it has been revealed that one of the plausible mechanisms for cycle cell disruptive effects of apigenin lies in RNAs. Apigenin modulates the transcription of regulatory mediators for the transition of cells from the G2 phase to the M phase by downregulation of cyclin mRNAs.^51^ Apigenin is also believed to cause upregulation of hsa‐miR‐215‐5p, a miRNA that regulates the expression of E2F transcription factors. E2F comprises multiple genes, which act as both cell cycle activators and inhibitors.^52^ Upregulation of hsa‐miR‐215‐5p has been demonstrated to be linked with the downregulation of activating E2F1/3, which results in cells remaining in quiescent condition.^20^ Apigenin exposure in a dose‐ and time‐dependent manner was linked to DNA damage and upregulation of p21 and p27, inhibitors of cyclin‐dependent kinase (CKD) in G1 and G2/M phases which also contributes to the stalling of cells in these cycles.^53^, ^54^

Apigenin has been shown to have favorable effects against malignant cell growth, invasion, and metastasis by modulating different stages of aberrant signaling pathways. In vitro experiments have shown that apigenin impacts regulatory molecules by repressing the STAT3 and NF‐κB, which are involved in the expression of adhesion molecules, enzymes, angiogenesis factors (e.g., VEGF‐C, MMP‐2/MMP‐9, E‐cadherin) and chemokines (e.g., CXCR4) responsible for malignant behaviors of CRC.^13^, ^39^, ^49^, ^55^ By acting on intracellular proteins, apigenin targets prosurvival regulators ERK and AKT leading to suppression of the tumoral cell growth and aggressiveness.^15^, ^22^, ^24^ Apigenin also suppresses the nuclear entry of β‐catenin and consecutively impairs Wnt downstream effector genes, which contributes to the tumoral cell invasion.^17^

Despite the many experiments done on CRC cell lines demonstrating the in vitro efficacy of apigenin, the effect of apigenin on CRC in vivo models has not yet been widely studied. The few current in vivo articles have mostly reported tumor size and body weight in colorectal adenocarcinoma mouse models. Mortality, being one of the most important outcomes in cancer research, has not been reported in most studies.

Our analysis of in vivo studies showed that apigenin can decrease tumor size when administered at doses higher than 30 mg/kg. Although due to the scarce number of experiments in the analysis, only the results of the 50 mg/kg dose subgroup can be relied upon.

Apigenin was not shown to affect the body weight of animal CRC models. However, only four articles had studied body weight as an outcome, and more studies are needed to assess the possible effect of apigenin on the body weight of animal CRC models.

Mortality was reported in two studies.^49^, ^56^ The studies assessed the efficacy of low and high‐dose protocols of apigenin on the mortality rate of animal models. Ai et al.^49^ demonstrated the mortality rate in groups of nontreated, low‐dose (200 mg/kg oral), and high‐dose (300 mg/kg, oral) apigenin‐treated animals were 17.2%, 22.4%, and 12.5%, respectively. Au et al.^56^ showed a mortality rate of 60%, 40% and 20% in nontreated animals, low‐dose treatment (0.025% dietary apigenin) and high‐dose treatment (0.1% dietary apigenin).

To the best of our knowledge, there has only been one systematic review on the effects of apigenin in cancer animal models. In their article, Singh et al.^57^ have demonstrated that in a pooled analysis of 25 studies on the effects of apigenin on various cancer animal models (three of which were studies on CRC animal models), apigenin reduces tumor volume, tumor weight, tumor number, and tumor load while having no significant effect on the animal's body weight. However, it should be noted that a limited number of studies were included for each of the cancer types. Our results further strengthen the observed effect of apigenin on the CRC cell lines and animal models by analyzing an increased number of studies.

## 5 LIMITATIONS AND FUTURE RECOMMENDATIONS

There are a few limitations to this systematic review and analysis. When interpreting the results of the current study, it should be kept in mind that different cell lines utilized in the included in vitro studies may not respond similarly to apigenin, as the discrete harboring mutations in various CRC cell lines may compromise the expression of apigenin targets and thus protecting them from its effects. Also, neither our review nor the review conducted by Singh et. al have provided any considerable results for the effects of apigenin on the mortality of CRC animal models. Considering that mortality is one of the most important outcomes in cancer research, future studies should report mortality as an outcome when studying the effects of apigenin on CRC animal models.

Apigenin has low solubility in water, which limits its therapeutic effects. It has been demonstrated that nanoparticles such as nanocrystals, apigenin‐loaded polymeric micelles, and apigenin liposomes can increase the bioavailability and therapeutic efficacy of apigenin against breast cancer.^58^ Future studies with improvements in drug delivery processes and enhanced bioavailability may further strengthen the antitumor properties of apigenin.

There are only a few clinical trials on the effects of apigenin in different clinical conditions. These studies have shown improvements in the condition of patients with anxiety and depression, Alzheimer's disease, and insomnia.^59^ We found only an observational analysis of human data on the effect of apigenin and flavonoids in general, which did not demonstrate any protective effect for apigenin in CRC and a few other cancers.^60^ Since the study was a secondary observational analysis, there is a need for further clinical trials to investigate the effect of apigenin on CRC cancer in humans. Our systematic review shows that the evidence for the effect of apigenin in in vivo studies is scarce, and more experiments in animal models need to be performed before the translation of the current evidence to clinical studies.

## 6 CONCLUSION

In summary, our results show that apigenin, through reducing cell viability, inducing growth inhibition, apoptosis, and cell cycle arrest and also by decreasing the tumor size can be considered as a possible adjuvant agent in the management of colorectal adenocarcinoma. However, further in vivo studies are needed to achieve a comprehensive conclusion on the possible effects of apigenin on CRC.

## AUTHOR CONTRIBUTIONS

**Koohyar Ahmadzadeh:** Data curation (equal); formal analysis (equal); investigation (equal); writing – original draft (equal). **Shayan Roshdi Dizaji:** Writing – original draft (equal). **Fatemeh Ramezani:** Writing – original draft (equal). **Farnad Imani:** Conceptualization (equal); methodology (equal); writing – original draft (equal). **Jebreil Shamseddin:** Writing – original draft (equal). **Arash Sarveazad:** Conceptualization (equal); data curation (equal); investigation (equal); methodology (equal); writing – original draft (equal). **Mahmoud Yousefifard:** Conceptualization (equal); data curation (equal); formal analysis (equal); investigation (equal); project administration (lead); supervision (lead); writing – original draft (equal).

## FUNDING INFORMATION

This study has been funded and supported by the Iran University of Medical Sciences (IUMS); Grant No. 99–1–49‐17478.

## CONFLICT OF INTEREST STATEMENT

The authors declare that they have no competing interests.

## ETHICS STATEMENT

Ethical approval was provided by the ethics committee of the Iran University of Medical Sciences (IR.IUMS.REC.1399.769).

## CONSENT

This study is a systematic review, and no individual patient data has been collected. This study was granted an exempt in this regard.

## Supporting information


Data S1.

Appendix S1.

## Data Availability

The gathered data can be shared at the request of qualified investigators with the purpose of replicating the procedures and results.

## References

[1] Bray F , Laversanne M , Sung H , et al. Global cancer statistics 2022: GLOBOCAN estimates of incidence and mortality worldwide for 36 cancers in 185 countries. CA Cancer J Clin. 2024;74(3):229‐263. 38572751 10.3322/caac.21834

[2] Anitha A , Maya S , Sivaram AJ , Mony U , Jayakumar R . Combinatorial nanomedicines for colon cancer therapy. Wiley Interdiscip Rev Nanomed Nanobiotechnol. 2016;8(1):151‐159. 26061225 10.1002/wnan.1353

[3] Jayaprakasha GK , Mandadi KK , Poulose SM , Jadegoud Y , Nagana Gowda GA , Patil BS . Inhibition of colon cancer cell growth and antioxidant activity of bioactive compounds from Poncirus trifoliata (L.) Raf. Bioorg Med Chem. 2007;15(14):4923‐4932. 17512744 10.1016/j.bmc.2007.04.044

[4] Boyle P , Leon ME . Epidemiology of colorectal cancer. Br Med Bull. 2002;64:1‐25. 12421722 10.1093/bmb/64.1.1

[5] Slattery ML , Potter JD , Coates A , et al. Plant foods and colon cancer: an assessment of specific foods and their related nutrients (United States). Cancer Causes Control. 1997;8(5):575‐590. 9242473 10.1023/a:1018490212481

[6] Santhosh R , Suriyanarayanan B . Plants: a source for new Antimycobacterial drugs. Planta Med. 2013;80(1):9‐21. 24218370 10.1055/s-0033-1350978

[7] Chidambara Murthy KN , Kim J , Vikram A , Patil BS . Differential inhibition of human colon cancer cells by structurally similar flavonoids of citrus. Food Chem. 2012;132(1):27‐34. 26434259 10.1016/j.foodchem.2011.10.014

[8] Fernández J , Silván B , Entrialgo‐Cadierno R , et al. Antiproliferative and palliative activity of flavonoids in colorectal cancer. Biomed Pharmacother. 2021;143:112241. 34649363 10.1016/j.biopha.2021.112241

[9] Peterson J , Dwyer J . Flavonoids: dietary occurrence and biochemical activity. Nutr Res. 1998;18(12):1995‐2018.

[10] Verma AK , Pratap R . The biological potential of flavones. Nat Prod Rep. 2010;27(11):1571‐1593. 20877900 10.1039/c004698c

[11] Budhraja A , Gao N , Zhang Z , et al. Apigenin induces apoptosis in human leukemia cells and exhibits anti‐leukemic activity *in vivo* . Mol Cancer Ther. 2012;11(1):132‐142. 22084167 10.1158/1535-7163.MCT-11-0343 PMC4430727

[12] Choi EJ , Kim G‐H . Apigenin induces apoptosis through a mitochondria/caspase‐pathway in human breast cancer MDA‐MB‐453 cells. J Clin Biochem Nutr. 2009;44(3):260‐265. 19430615 10.3164/jcbn.08-230 PMC2675027

[13] Wang L , Kuang L , Hitron JA , et al. Apigenin suppresses migration and invasion of transformed cells through down‐regulation of C‐X‐C chemokine receptor 4 expression. Toxicol Appl Pharmacol. 2013;272(1):108‐116. 23743303 10.1016/j.taap.2013.05.028 PMC3823051

[14] Shukla S , Gupta S . Apigenin: a promising molecule for cancer prevention. Pharm Res. 2010;27(6):962‐978. 20306120 10.1007/s11095-010-0089-7 PMC2874462

[15] Shao H , Jing K , Mahmoud E , Huang H , Fang X , Yu C . Apigenin sensitizes colon cancer cells to antitumor activity of ABT‐263. Mol Cancer Ther. 2013;12(12):2640‐2650. 24126433 10.1158/1535-7163.MCT-13-0066 PMC3871201

[16] Chung CS , Jiang Y , Cheng D , Birt DF . Impact of adenomatous polyposis coli (APC) tumor supressor gene in human colon cancer cell lines on cell cycle arrest by apigenin. Mol Carcinog. 2007;46(9):773‐782. 17620292 10.1002/mc.20306

[17] Xu M , Wang S , Song Y , Yao J , Huang K , Zhu X . Apigenin suppresses colorectal cancer cell proliferation, migration and invasion via inhibition of the Wnt/β‐catenin signaling pathway. Oncol Lett. 2016;11(5):3075‐3080. 27123066 10.3892/ol.2016.4331 PMC4840993

[18] Banerjee K , Banerjee S , Mandal M . Enhanced chemotherapeutic efficacy of apigenin liposomes in colorectal cancer based on flavone‐membrane interactions. J Colloid Interface Sci. 2017;491:98‐110. 28012918 10.1016/j.jcis.2016.12.025

[19] Buhagiar JA , Bertoli A , Camilleri‐Podesta MT , Pistelli L . *In vitro* apoptotic bioactivity of flavonoids from *Astragalus Verrucosus* Moris. Nat Prod Commun. 2008;3(12):1934578X0800301.

[20] Cheng Y , Han X , Mo F , et al. Apigenin inhibits the growth of colorectal cancer through down‐regulation of E2F1/3 by miRNA‐215‐5p. Phytomedicine. 2021;89:153603. 34175590 10.1016/j.phymed.2021.153603

[21] Cho Y , Choi M‐Y . Inhibitory effects of flavonoids on growth of HT‐29 human colon cancer cells. J Korean Soc Food Sci Nutr. 2015;44(3):338‐346.

[22] Chunhua L , Donglan L , Xiuqiong F , et al. Apigenin up‐regulates transgelin and inhibits invasion and migration of colorectal cancer through decreased phosphorylation of AKT. J Nutr Biochem. 2013;24(10):1766‐1775. 23773626 10.1016/j.jnutbio.2013.03.006

[23] Cicek M , Unsal V , Emre A , Doganer A . Investigation of the effects of Apigenin, a possible therapeutic agent, on cytotoxic and SWH pathway in colorectal cancer (HT29) cells. Adv Pharm Bull. 2023;13(1):188‐195. 36721804 10.34172/apb.2023.020 PMC9871274

[24] Dai J , Van Wie PG , Fai LY , et al. Downregulation of NEDD9 by apigenin suppresses migration, invasion, and metastasis of colorectal cancer cells. Toxicol Appl Pharmacol. 2016;311:106‐112. 27664007 10.1016/j.taap.2016.09.016 PMC5759047

[25] Farah M , Parhar K , Moussavi M , Eivemark S , Salh B . 5,6‐Dichloro‐ribifuranosylbenzimidazole‐ and apigenin‐induced sensitization of colon cancer cells to TNF‐α‐mediated apoptosis. Am J Physiol Gastrointest Liver Physiol. 2003;285(5):G919‐G928. 12842827 10.1152/ajpgi.00205.2003

[26] Hong S , Dia VP , Baek SJ , Zhong Q . Nanoencapsulation of apigenin with whey protein isolate: physicochemical properties, in vitro activity against colorectal cancer cells, and bioavailability. Lebensm Wiss Technol. 2022;154:112751. 34840350 10.1016/j.lwt.2021.112751 PMC8612601

[27] Iizumi Y , Oishi M , Taniguchi T , Goi W , Sowa Y , Sakai T . The flavonoid Apigenin downregulates CDK1 by directly targeting ribosomal protein S9. PLoS One. 2013;8(8):e73219. 24009741 10.1371/journal.pone.0073219 PMC3756953

[28] Kim D‐H , Lee J‐T , Lee I‐K , Ha J‐H . Comparative anticancer effects of flavonoids and diazepam in cultured cancer cells. Biol Pharm Bull. 2008;31(2):255‐259. 18239283 10.1248/bpb.31.255

[29] Klampfer L , Huang J , Sasazuki T , Shirasawa S , Augenlicht L . Oncogenic Ras promotes butyrate‐induced apoptosis through inhibition of gelsolin expression. J Biol Chem. 2004;279(35):36680‐36688. 15213223 10.1074/jbc.M405197200

[30] Lee S‐W , Lee J‐T , Lee M‐G , et al. In vitro antiproliferative characteristics of flavonoids and diazepam on SNU‐C4 colorectal adenocarcinoma cells. J Nat Med. 2009;63(2):124‐129. 19050992 10.1007/s11418-008-0300-x

[31] Lee Y , Sung B , Kang YJ , et al. Apigenin‐induced apoptosis is enhanced by inhibition of autophagy formation in HCT116 human colon cancer cells. Int J Oncol. 2014;44(5):1599‐1606. 24626522 10.3892/ijo.2014.2339

[32] Richter M , Ebermann R , Marian B . Quercetin‐induced apoptosis in colorectal tumor cells: possible role of EGF receptor signaling. Nutr Cancer. 1999;34(1):88‐99. 10453447 10.1207/S15327914NC340113

[33] Sain A , Khamrai D , Kandasamy T , Naskar D . Apigenin exerts anti‐cancer effects in colon cancer by targeting HSP90AA1. J Biomol Struct Dyn. 2023;1‐13. 10.1080/07391102.2023.2299305 38157250

[34] Shan S , Shi J , Yang P , et al. Apigenin restrains colon cancer cell proliferation via targeted blocking of pyruvate kinase M2‐dependent glycolysis. J Agric Food Chem. 2017;65(37):8136‐8144. 28829588 10.1021/acs.jafc.7b02757

[35] Shi J , Ji X , Shan S , Zhao M , Bi C , Li Z . The interaction between apigenin and PKM2 restrains progression of colorectal cancer. J Nutr Biochem. 2023;121:109430. 37597817 10.1016/j.jnutbio.2023.109430

[36] Simsek EN , Uysal T . In vitro investigation of cytotoxic and apoptotic effects of Cynara L. species in colorectal cancer cells. Asian Pac J Cancer Prev. 2013;14(11):6791‐6795. 24377607 10.7314/apjcp.2013.14.11.6791

[37] Smiljkovic M , Stanisavljevic D , Stojkovic D , et al. Apigenin‐7‐O‐glucoside versus apigenin: insight into the modes of anticandidal and cytotoxic actions. EXCLI J. 2017;16:795‐807. 28827996 10.17179/excli2017-300 PMC5547395

[38] Takagaki N , Sowa Y , Oki T , Nakanishi R , Yogosawa S , Sakai T . Apigenin induces cell cycle arrest and p21/WAFl expression in a p53‐independent pathway. Int J Oncol. 2005;26(1):185‐189. 15586239

[39] Tong J , Shen Y , Zhang Z , Hu Y , Zhang X , Han L . Apigenin inhibits epithelial‐mesenchymal transition of human colon cancer cells through NF‐κB/snail signaling pathway. Biosci Rep. 2019;39(5):BSR20190452. 30967496 10.1042/BSR20190452 PMC6522743

[40] Turktekin M , Konac E , Onen HI , Alp E , Yilmaz A , Menevse S . Evaluation of the effects of the flavonoid Apigenin on apoptotic pathway gene expression on the colon cancer cell line (HT29). J Med Food. 2011;14(10):1107‐1117. 21548803 10.1089/jmf.2010.0208

[41] Wang W , Heideman L , Chung CS , Pelling JC , Koehler KJ , Birt DF . Cell‐cycle arrest at G2/M and growth inhibition by Apigenin in human colon carcinoma cell lines. Mol Carcinog. 2000;28(2):102‐110. 10900467

[42] Wang W , VanAlstyne PC , Irons KA , Chen S , Stewart JW , Birt DF . Individual and interactive effects of Apigenin analogs on G2/M cell‐cycle arrest in human colon carcinoma cell lines. Nutr Cancer. 2004;48(1):106‐114. 15203384 10.1207/s15327914nc4801_14

[43] Wang B , Zhao X‐H . Apigenin induces both intrinsic and extrinsic pathways of apoptosis in human colon carcinoma HCT‐116 cells. Oncol Rep. 2016;37(2):1132‐1140. 27959417 10.3892/or.2016.5303

[44] Wang B , Zhao X . Four in vitro activities of apigenin to human colorectal carcinoma cells susceptible to air‐oxidative and heating treatments. Emir J Food Agric. 2017;29(1):69‐77.

[45] Wang J , Li T , Zang L , et al. Apigenin inhibits human SW620 cell growth by targeting polyamine catabolism. Evid Based Complement Alternat Med. 2017;2017:3684581. 28572828 10.1155/2017/3684581 PMC5442336

[46] Yang C , Song J , Hwang S , Choi J , Song G , Lim W . Apigenin enhances apoptosis induction by 5‐fluorouracil through regulation of thymidylate synthase in colorectal cancer cells. Redox Biol. 2021;47:102144. 34562873 10.1016/j.redox.2021.102144 PMC8476449

[47] Zhang X , Zhang W , Chen F , Lu Z . Combined effect of chrysin and apigenin on inhibiting the development and progression of colorectal cancer by suppressing the activity of P38‐MAPK/AKT pathway. IUBMB Life. 2021;73(5):774‐783. 33625784 10.1002/iub.2456

[48] Zhong Y , Krisanapun C , Lee S‐H , et al. Molecular targets of apigenin in colorectal cancer cells: involvement of p21, NAG‐1 and p53. Eur J Cancer. 2010;46(18):3365‐3374. 20709524 10.1016/j.ejca.2010.07.007 PMC2988105

[49] Ai X‐Y , Qin Y , Liu H‐J , et al. Apigenin inhibits colonic inflammation and tumorigenesis by suppressing STAT3‐NF‐κB signaling. Oncotarget. 2017;8(59):100216‐100226. 29245972 10.18632/oncotarget.22145 PMC5725014

[50] Bian S , Wan H , Liao X , Wang W . Inhibitory effects of Apigenin on tumor carcinogenesis by altering the gut microbiota. Mediat Inflamm. 2020;2020(1):7141970. 10.1155/2020/7141970 PMC7559228 33082711

[51] Hnit SST , Yao M , Xie C , et al. Apigenin impedes cell cycle progression at G2 phase in prostate cancer cells. Discov Oncol. 2022;13(1):44. 35670862 10.1007/s12672-022-00505-1 PMC9174405

[52] Zhu W , Giangrande PH , Nevins JR . E2Fs link the control of G1/S and G2/M transcription. EMBO J. 2004;23(23):4615‐4626. 15510213 10.1038/sj.emboj.7600459 PMC533046

[53] Oishi M , Iizumi Y , Taniguchi T , Goi W , Miki T , Sakai T . Apigenin sensitizes prostate cancer cells to Apo2L/TRAIL by targeting adenine nucleotide Translocase‐2. PLoS One. 2013;8(2):e55922. 23431365 10.1371/journal.pone.0055922 PMC3576345

[54] Rahmani AH , Alsahli MA , Almatroudi A , et al. The potential role of Apigenin in cancer prevention and treatment. Molecules. 2022;27(18):6051. 36144783 10.3390/molecules27186051 PMC9505045

[55] Liu L‐Z , Fang J , Zhou Q , Hu X , Shi X , Jiang B‐H . Apigenin inhibits expression of vascular endothelial growth factor and angiogenesis in human lung cancer cells: implication of chemoprevention of lung cancer. Mol Pharmacol. 2005;68(3):635‐643. 15947208 10.1124/mol.105.011254

[56] Au A , Li B , Wang W , Roy H , Koehler K , Birt D . Effect of dietary Apigenin on colonic ornithine decarboxylase activity, aberrant crypt foci formation, and tumorigenesis in different experimental models. Nutr Cancer. 2006;54(2):243‐251. 16898869 10.1207/s15327914nc5402_11

[57] Singh D , Gupta M , Sarwat M , Siddique HR . Apigenin in cancer prevention and therapy: a systematic review and meta‐analysis of animal models. Crit Rev Oncol Hematol. 2022;176:103751. 35752426 10.1016/j.critrevonc.2022.103751

[58] Adel M , Zahmatkeshan M , Akbarzadeh A , et al. Chemotherapeutic effects of Apigenin in breast cancer: preclinical evidence and molecular mechanisms; enhanced bioavailability by nanoparticles. Biotechnol Rep (Amst). 2022;34:e00730. 35686000 10.1016/j.btre.2022.e00730 PMC9171451

[59] Salehi B , Venditti A , Sharifi‐Rad M , et al. The therapeutic potential of Apigenin. Int J Mol Sci. 2019;20(6):1305. 30875872 10.3390/ijms20061305 PMC6472148

[60] Wang L , Lee I‐M , Zhang SM , Blumberg JB , Buring JE , Sesso HD . Dietary intake of selected flavonols, flavones, and flavonoid‐rich foods and risk of cancer in middle‐aged and older women. Am J Clin Nutr. 2009;89(3):905‐912. 19158208 10.3945/ajcn.2008.26913 PMC2667658

