# Supplementary material for: Potential therapeutic effects of apigenin for colorectal adenocarcinoma: A systematic review and meta‐analysis
Source: Cancer Med. 2024 Sep 10;13(17):e70171. doi: 10.1002/cam4.70171 (PMC11386296; doi:10.1002/cam4.70171)
Supplement: Supplementary file 1 — Data S1. [file CAM4-13-e70171-s001.docx]

# Supplementary material 1: Search queries for databases

# Medline (via PubMed):

# “Colorectal Neoplasms”[mh] OR “Adenomatous Polyposis Coli”[mh] OR “Gardner Syndrome”[mh] OR “Colonic Neoplasms”[mh] OR “Colitis-Associated Neoplasms”[mh] OR “Sigmoid Neoplasms”[mh] OR “Colorectal Neoplasms, Hereditary Nonpolyposis”[mh] OR “Rectal Neoplasms”[mh] OR “Anus Neoplasms”[mh] OR “Anal Gland Neoplasms”[mh] OR “Cecal Neoplasms”[mh] OR Colorectal Neoplasm[tiab] OR Colorectal Tumor[tiab] OR Colorectal Cancer[tiab] OR Colorectal Carcinoma[tiab] OR Familial Polyposis Syndrome[tiab] OR Adenomatous Polyposis of the Colon[tiab] OR Familial Adenomatous Polyposis[tiab] OR Familial Multiple Polyposi[tiab] OR Familial Polyposis of the Colon[tiab] OR Familial Multiple Polyposis[tiab] OR Familial Multiple Polyposis Syndrome[tiab] OR Familial Polyposis Syndrome[tiab] OR Myh-Associated Polyposis[tiab] OR Myh Associated Polyposis[tiab] OR Polyposis Coli[tiab] OR Polyposis Colus[tiab] OR Familial Intestinal Polyposis[tiab] OR Adenomatous Intestinal Polyposis[tiab] OR Gardner Syndrome[tiab] OR Colonic Neoplasm[tiab] OR Colon Neoplasm[tiab] OR Cancer of Colon[tiab] OR Colon Cancer[tiab] OR Cancer of the Colon[tiab] OR Colonic Cancer[tiab] OR Colon Adenocarcinoma[tiab] OR Colitis Associated Neoplasm[tiab] OR Colitis-Associated Neoplasm[tiab] OR Colitis-Associated Colorectal Cancer[tiab] OR Colitis Associated Colorectal Cancer[tiab] OR Colitis-Associated Cancer[tiab] OR Colitis Associated Cancer[tiab] OR Colitis-Associated Colon Cancer[tiab] OR Colitis Associated Colon Cancer[tiab] OR Sigmoid Neoplasm[tiab] OR Sigmoid Colon Neoplasm[tiab] OR Sigmoid Cancer[tiab] OR Sigmoidal Cancer[tiab] OR Sigmoid Colon Cancer[tiab] OR Cancer of Sigmoid[tiab] OR Cancer of the Sigmoid[tiab] OR Lynch Syndrome[tiab] OR Lynch Cancer [tiab] OR Rectal Neoplasm[tiab] OR Rectum Neoplasm[tiab] OR Rectal Tumor[tiab] OR Cancer of Rectum[tiab] OR Rectum Cancer[tiab] OR Rectal Cancer[tiab] OR Cancer of the Rectum[tiab] OR Anal Neoplasm[tiab] OR Anus Neoplasm[tiab] OR Anal Cancer[tiab] OR Cancer of the Anus[tiab] OR Cancer of Anus[tiab] OR Anus Cancer[tiab] OR Perianal Gland Neoplasm[tiab] OR Anal Gland Neoplasm[tiab] OR colorectal neoplasia[tiab] OR colorectal tumour[tiab] OR neoplasma recti[tiab] OR pararectal tumor[tiab] OR pararectal tumour [tiab] OR rectal tumour [tiab] OR retrorectal tumor[tiab] OR retrorectal tumour[tiab] OR tumor recti[tiab] OR tumour recti[tiab] OR adenocarcinoma recti[tiab] OR carcinoma adenomatosum recti[tiab] OR carcinoma recti[tiab] OR rectal adenocarcinoma[tiab] OR rectum adenocarcinoma[tiab] OR rectum ampulla carcinoma[tiab] OR colon tumour[tiab] OR sigmoid tumour[tiab] OR mesocolon tumour[tiab] OR carcinoma coli[tiab] OR colonic carcinoma[tiab] OR rectosigmoid adenocarcinoma[tiab] OR colon metastasis[tiab] OR Cecal Neoplasm[tiab] OR Cancer of Cecum[tiab] OR Cecal Cancer[tiab] OR Cancer of the Cecum[tiab] OR caecal tumor [tiab] OR caecal tumour [tiab] OR cecal tumor[tiab] OR cecal tumour[tiab] OR cecum tumour[tiab] OR coecum tumor[tiab] OR coecum tumour[tiab]

# “Apigenin”[mh] OR Apigenin[tiab]

# #1 AND #2

**Embase:**

1. 'colorectal tumor'/exp OR 'colorectal adenoma'/exp OR ‘colorectal tumor’/exp OR ‘rectum adenoma’/exp OR ‘rectum cancer’/exp OR ‘rectum tumor’/exp OR ‘rectum carcinoma’/exp OR ‘colon tumor’/exp OR ‘colon adenoma’/exp OR ‘colon cancer’/exp OR ‘colon carcinogenesis’/exp OR ‘colon carcinoma’/exp OR ‘colorectal cancer’/exp OR ‘experimental colon cancer’/exp OR ‘metastatic colon cancer’/exp OR ‘sigmoid cancer’/exp OR ‘colon adenocarcinoma’/exp OR ‘sigmoid carcinoma’/exp OR ‘cecum tumor’/exp OR ‘Colorectal Neoplasm’:ab,ti OR ‘Colorectal Tumor’:ab,ti OR ‘Colorectal Cancer’:ab,ti OR ‘Colorectal Carcinoma’:ab,ti OR ‘Familial Polyposis Syndrome’:ab,ti OR ‘Adenomatous Polyposis of the Colon’:ab,ti OR ‘Familial Adenomatous Polyposis’:ab,ti OR ‘Familial Multiple Polyposi’:ab,ti OR ‘Familial Polyposis of the Colon’:ab,ti OR ‘Familial Multiple Polyposis’:ab,ti OR ‘Familial Multiple Polyposis Syndrome’:ab,ti OR ‘Familial Polyposis Syndrome’:ab,ti OR ‘Myh-Associated Polyposis’:ab,ti OR ‘Myh Associated Polyposis’:ab,ti OR ‘Polyposis Coli’:ab,ti OR ‘Polyposis Colus’:ab,ti OR ‘Familial Intestinal Polyposis’:ab,ti OR ‘Adenomatous Intestinal Polyposis’:ab,ti OR ‘Gardner Syndrome’:ab,ti OR ‘Colonic Neoplasm’:ab,ti OR ‘Colon Neoplasm’:ab,ti OR ‘Cancer of Colon’:ab,ti OR ‘Colon Cancer’:ab,ti OR ‘Cancer of the Colon’:ab,ti OR ‘Colonic Cancer’:ab,ti OR ‘Colon Adenocarcinoma’:ab,ti OR ‘Colitis Associated Neoplasm’:ab,ti OR ‘Colitis-Associated Neoplasm’:ab,ti OR ‘Colitis-Associated Colorectal Cancer’:ab,ti OR ‘Colitis Associated Colorectal Cancer’:ab,ti OR ‘Colitis-Associated Cancer’:ab,ti OR ‘Colitis Associated Cancer’:ab,ti OR ‘Colitis-Associated Colon Cancer’:ab,ti OR ‘Colitis Associated Colon Cancer’:ab,ti OR ‘Sigmoid Neoplasm’:ab,ti OR ‘Sigmoid Colon Neoplasm’:ab,ti OR ‘Sigmoid Cancer’:ab,ti OR ‘Sigmoidal Cancer’:ab,ti OR ‘Sigmoid Colon Cancer’:ab,ti OR ‘Cancer of Sigmoid’:ab,ti OR ‘Cancer of the Sigmoid’:ab,ti OR ‘Lynch Syndrome’:ab,ti OR ‘Lynch Cancer ‘:ab,ti OR ‘Rectal Neoplasm’:ab,ti OR ‘Rectum Neoplasm’:ab,ti OR ‘Rectal Tumor’:ab,ti OR ‘Cancer of Rectum’:ab,ti OR ‘Rectum Cancer’:ab,ti OR ‘Rectal Cancer’:ab,ti OR ‘Cancer of the Rectum’:ab,ti OR ‘Anal Neoplasm’:ab,ti OR ‘Anus Neoplasm’:ab,ti OR ‘Anal Cancer’:ab,ti OR ‘Cancer of the Anus’:ab,ti OR ‘Cancer of Anus’:ab,ti OR ‘Anus Cancer’:ab,ti OR ‘Perianal Gland Neoplasm’:ab,ti OR ‘Anal Gland Neoplasm’:ab,ti OR ‘colorectal neoplasia’:ab,ti OR ‘colorectal tumour’:ab,ti OR ‘neoplasma recti’:ab,ti OR ‘pararectal tumor’:ab,ti OR ‘pararectal tumour ‘:ab,ti OR ‘rectal tumour‘:ab,ti OR ‘retrorectal tumor’:ab,ti OR ‘retrorectal tumour’:ab,ti OR ‘tumor recti’:ab,ti OR ‘tumour recti’:ab,ti OR ‘adenocarcinoma recti’:ab,ti OR ‘carcinoma adenomatosum recti’:ab,ti OR ‘carcinoma recti’:ab,ti OR ‘rectal adenocarcinoma’:ab,ti OR ‘rectum adenocarcinoma’:ab,ti OR ‘rectum ampulla carcinoma’:ab,ti OR ‘colon tumour’:ab,ti OR ‘sigmoid tumour’:ab,ti OR ‘mesocolon tumour’:ab,ti OR ‘carcinoma coli’:ab,ti OR ‘colonic carcinoma’:ab,ti OR ‘rectosigmoid adenocarcinoma’:ab,ti OR ‘colon metastasis’:ab,ti OR ‘Cecal Neoplasm’:ab,ti OR ‘Cancer of Cecum’:ab,ti OR ‘Cecal Cancer’:ab,ti OR ‘Cancer of the Cecum’:ab,ti OR ‘caecal tumor ‘:ab,ti OR ‘caecal tumour ‘:ab,ti OR ‘cecal tumor’:ab,ti OR ‘cecal tumour’:ab,ti OR ‘cecum tumour’:ab,ti OR ‘coecum tumor’:ab,ti OR ‘coecum tumour’:ab,ti
2. ‘Apigenin’/exp OR ‘Apigenin’:ab,ti

#1 AND #2

**Scopus:**

1. TITLE-ABS-KEY(“Colorectal Neoplasm” OR “Colorectal Tumor” OR “Colorectal Cancer” OR “Colorectal Carcinoma” OR “Familial Polyposis Syndrome” OR “Adenomatous Polyposis of the Colon” OR “Familial Adenomatous Polyposis” OR “Familial Multiple Polyposi” OR “Familial Polyposis of the Colon” OR “Familial Multiple Polyposis” OR “Familial Multiple Polyposis Syndrome” OR “Familial Polyposis Syndrome” OR “Myh-Associated Polyposis” OR “Myh Associated Polyposis” OR “Polyposis Coli” OR “Polyposis Colus” OR “Familial Intestinal Polyposis” OR “Adenomatous Intestinal Polyposis” OR “Gardner Syndrome” OR “Colonic Neoplasm” OR “Colon Neoplasm” OR “Cancer of Colon” OR “Colon Cancer” OR “Cancer of the Colon” OR “Colonic Cancer” OR “Colon Adenocarcinoma” OR “Colitis Associated Neoplasm” OR “Colitis-Associated Neoplasm” OR “Colitis-Associated Colorectal Cancer” OR “Colitis Associated Colorectal Cancer” OR “Colitis-Associated Cancer” OR “Colitis Associated Cancer” OR “Colitis-Associated Colon Cancer” OR “Colitis Associated Colon Cancer” OR “Sigmoid Neoplasm” OR “Sigmoid Colon Neoplasm” OR “Sigmoid Cancer” OR “Sigmoidal Cancer” OR “Sigmoid Colon Cancer” OR “Cancer of Sigmoid” OR “Cancer of the Sigmoid” OR “Lynch Syndrome” OR “Lynch Cancer “ OR “Rectal Neoplasm” OR “Rectum Neoplasm” OR “Rectal Tumor” OR “Cancer of Rectum” OR “Rectum Cancer” OR “Rectal Cancer” OR “Cancer of the Rectum” OR “Anal Neoplasm” OR “Anus Neoplasm” OR “Anal Cancer” OR “Cancer of the Anus” OR “Cancer of Anus” OR “Anus Cancer” OR “Perianal Gland Neoplasm” OR “Anal Gland Neoplasm” OR “colorectal neoplasia” OR “colorectal tumour” OR “neoplasma recti” OR “pararectal tumor” OR “pararectal tumour” OR “rectal tumour” OR “retrorectal tumor” OR “retrorectal tumour” OR “tumor recti” OR “tumour recti” OR “adenocarcinoma recti” OR “carcinoma adenomatosum recti” OR “carcinoma recti” OR “rectal adenocarcinoma” OR “rectum adenocarcinoma” OR “rectum ampulla carcinoma” OR “colon tumour” OR “sigmoid tumour” OR “mesocolon tumour” OR “carcinoma coli” OR “colonic carcinoma” OR “rectosigmoid adenocarcinoma” OR “colon metastasis” OR “Cecal Neoplasm” OR “Cancer of Cecum” OR “Cecal Cancer” OR “Cancer of the Cecum” OR “caecal tumor” OR “caecal tumour” OR “cecal tumor” OR “cecal tumour” OR “cecum tumour” OR “coecum tumor” OR “coecum tumour”)
2. TITLE-ABS-KEY(“Apigenin”)

#1 AND #2

**Web of Science:**

1. TS=(“Colorectal Neoplasm” OR “Colorectal Tumor” OR “Colorectal Cancer” OR “Colorectal Carcinoma” OR “Familial Polyposis Syndrome” OR “Adenomatous Polyposis of the Colon” OR “Familial Adenomatous Polyposis” OR “Familial Multiple Polyposi” OR “Familial Polyposis of the Colon” OR “Familial Multiple Polyposis” OR “Familial Multiple Polyposis Syndrome” OR “Familial Polyposis Syndrome” OR “Myh-Associated Polyposis” OR “Myh Associated Polyposis” OR “Polyposis Coli” OR “Polyposis Colus” OR “Familial Intestinal Polyposis” OR “Adenomatous Intestinal Polyposis” OR “Gardner Syndrome” OR “Colonic Neoplasm” OR “Colon Neoplasm” OR “Cancer of Colon” OR “Colon Cancer” OR “Cancer of the Colon” OR “Colonic Cancer” OR “Colon Adenocarcinoma” OR “Colitis Associated Neoplasm” OR “Colitis-Associated Neoplasm” OR “Colitis-Associated Colorectal Cancer” OR “Colitis Associated Colorectal Cancer” OR “Colitis-Associated Cancer” OR “Colitis Associated Cancer” OR “Colitis-Associated Colon Cancer” OR “Colitis Associated Colon Cancer” OR “Sigmoid Neoplasm” OR “Sigmoid Colon Neoplasm” OR “Sigmoid Cancer” OR “Sigmoidal Cancer” OR “Sigmoid Colon Cancer” OR “Cancer of Sigmoid” OR “Cancer of the Sigmoid” OR “Lynch Syndrome” OR “Lynch Cancer “ OR “Rectal Neoplasm” OR “Rectum Neoplasm” OR “Rectal Tumor” OR “Cancer of Rectum” OR “Rectum Cancer” OR “Rectal Cancer” OR “Cancer of the Rectum” OR “Anal Neoplasm” OR “Anus Neoplasm” OR “Anal Cancer” OR “Cancer of the Anus” OR “Cancer of Anus” OR “Anus Cancer” OR “Perianal Gland Neoplasm” OR “Anal Gland Neoplasm” OR “colorectal neoplasia” OR “colorectal tumour” OR “neoplasma recti” OR “pararectal tumor” OR “pararectal tumour“ OR “rectal tumour“ OR “retrorectal tumor” OR “retrorectal tumour” OR “tumor recti” OR “tumour recti” OR “adenocarcinoma recti” OR “carcinoma adenomatosum recti” OR “carcinoma recti” OR “rectal adenocarcinoma” OR “rectum adenocarcinoma” OR “rectum ampulla carcinoma” OR “colon tumour” OR “sigmoid tumour” OR “mesocolon tumour” OR “carcinoma coli” OR “colonic carcinoma” OR “rectosigmoid adenocarcinoma” OR “colon metastasis” OR “Cecal Neoplasm” OR “Cancer of Cecum” OR “Cecal Cancer” OR “Cancer of the Cecum” OR “caecal tumor“ OR “caecal tumour“ OR “cecal tumor” OR “cecal tumour” OR “cecum tumour” OR “coecum tumor” OR “coecum tumour”)
2. TS=(“Apigenin”)

#1 AND #2
