# Supplementary material for: Potential therapeutic effects of apigenin for colorectal adenocarcinoma: A systematic review and meta‐analysis
Source: Cancer Med. 2024 Sep 10;13(17):e70171. doi: 10.1002/cam4.70171 (PMC11386296; doi:10.1002/cam4.70171)
Supplement: Supplementary file 2 — Appendix S1. [file CAM4-13-e70171-s002.docx]

Dose-dependent effects of Apigenin on cell viability, growth inhibition and apoptosis of CRC cell lines.

This supplementary material includes the results of the analysis for Dose-dependent effects of Apigenin on cell viability, growth inhibition and apoptosis of CRC cell lines.

Any missing dose groups (e.g: dose group for between 60.1 to 70 μM in cell viability) is due to no reported data in the included articles.

Table of Contents

[Cell Viability 3](#_Toc112189619)

[Supplementary Figure1. Effect of apigenin on cell viability in CRC cell lines with administered dosages ≤ 10 μM 4](#_Toc112189620)

[Supplementary Figure 2. Effect of apigenin on cell viability in CRC cell lines with administered dosages between 10.1 and 20 μM 5](#_Toc112189621)

[Supplementary Figure 3. Effect of apigenin on cell viability in CRC cell lines with administered dosages between 20.1 and 30 μM 6](#_Toc112189622)

[Supplementary Figure 4. Effect of apigenin on cell viability in CRC cell lines with administered dosages between 30.1 and 40 μM 7](#_Toc112189623)

[Supplementary Figure 5. Effect of apigenin on cell viability in CRC cell lines with administered dosages between 40.1 and 50 μM 8](#_Toc112189624)

[Supplementary Figure 6. Effect of apigenin on cell viability in CRC cell lines with administered dosages between 50.1 and 60 μM 9](#_Toc112189625)

[Supplementary Figure 7. Effect of apigenin on cell viability in CRC cell lines with administered dosages between 70.1 and 80 μM 10](#_Toc112189626)

[Supplementary Figure 8. Effect of apigenin on cell viability of CRC cell lines with administered dosages of 90.1 to 100 μM 11](#_Toc112189627)

[Supplementary Figure 9. Effect of apigenin on cell viability in CRC cell lines with administered dosages between of 120.1 and 160 μM 12](#_Toc112189628)

[Supplementary Figure 10. Effect of apigenin on cell viability in CRC cell lines with administered dosages with dosages of 200 μM 13](#_Toc112189629)

[Supplementary Figure 11. Effect of apigenin on cell viability in CRC cell lines with administered dosages between 200.1 and 1000 μM 14](#_Toc112189630)

[Supplementary Figure 12. Effect of apigenin on cell viability in CRC cell lines with administered dosages more than 1000.1 μM 15](#_Toc112189631)

[Growth Inhibition 16](#_Toc112189632)

[Supplementary Figure 13. Effect of apigenin on growth inhibition in CRC cell lines with administered dosages ≤ 20 μM 16](#_Toc112189633)

[Supplementary Figure 14. Effect of apigenin on growth inhibition in CRC cell lines with administered dosages between 20.1 to 40 μM 17](#_Toc112189634)

[Supplementary Figure 15. Effect of apigenin on growth inhibition with dosages between 60.1 to 80 μM 18](#_Toc112189635)

[Supplementary Figure 16. Effect of apigenin on growth inhibition with dosages of 120.1 and 160 μM 19](#_Toc112189636)

[Apoptosis 20](#_Toc112189637)

[Supplementary Figure 17. Effect of apigenin on apoptosis with dosages ≤ 10 μM 20](#_Toc112189638)

[Supplementary Figure 18. Effect of apigenin on apoptosis with dosages between 10.1 to 100 μM 21](#_Toc112189639)

# **Cell Viability**

# **
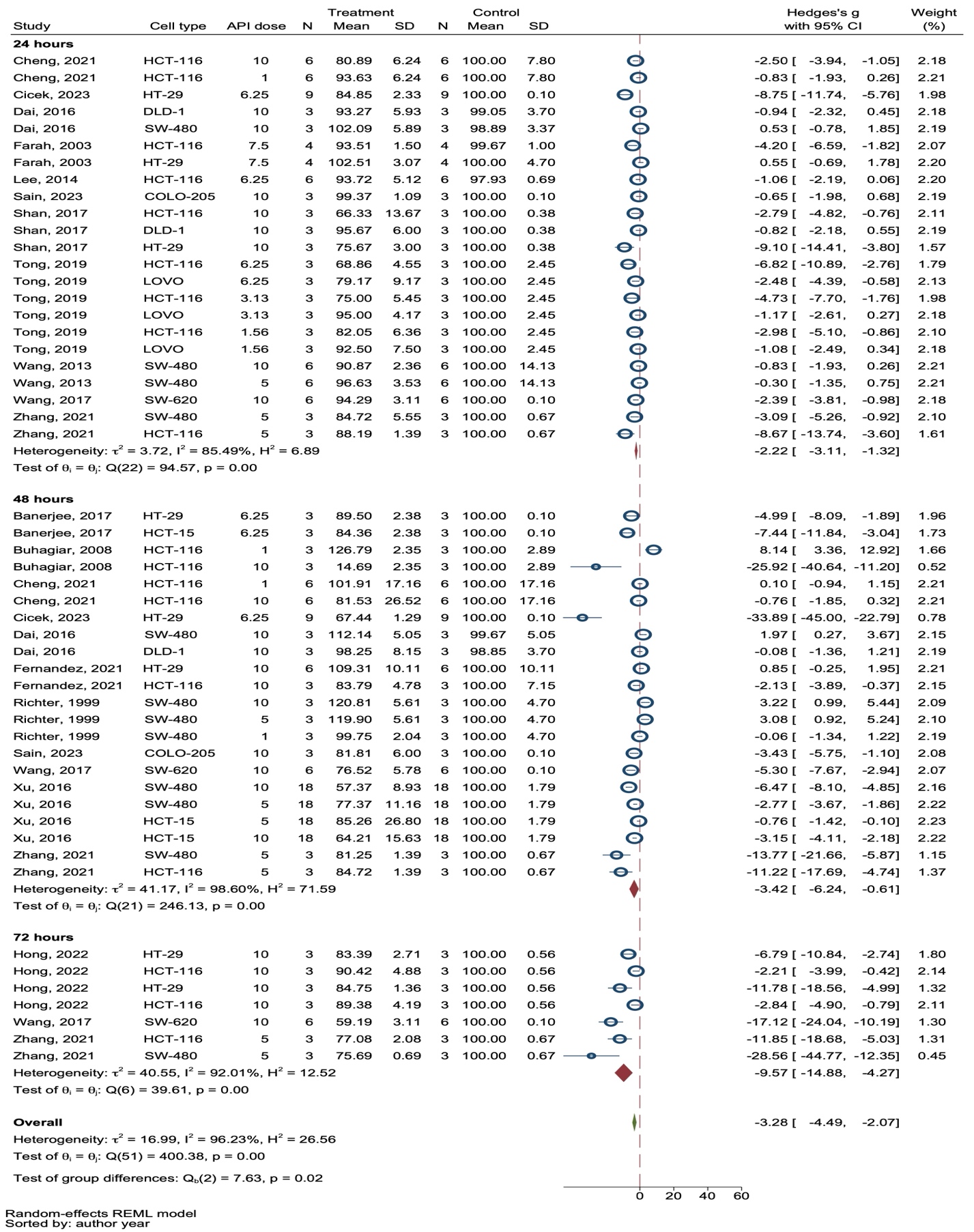
**

## Supplementary Figure1. Effect of apigenin on cell viability in CRC cell lines with administered dosages ≤ 10 μM.

##
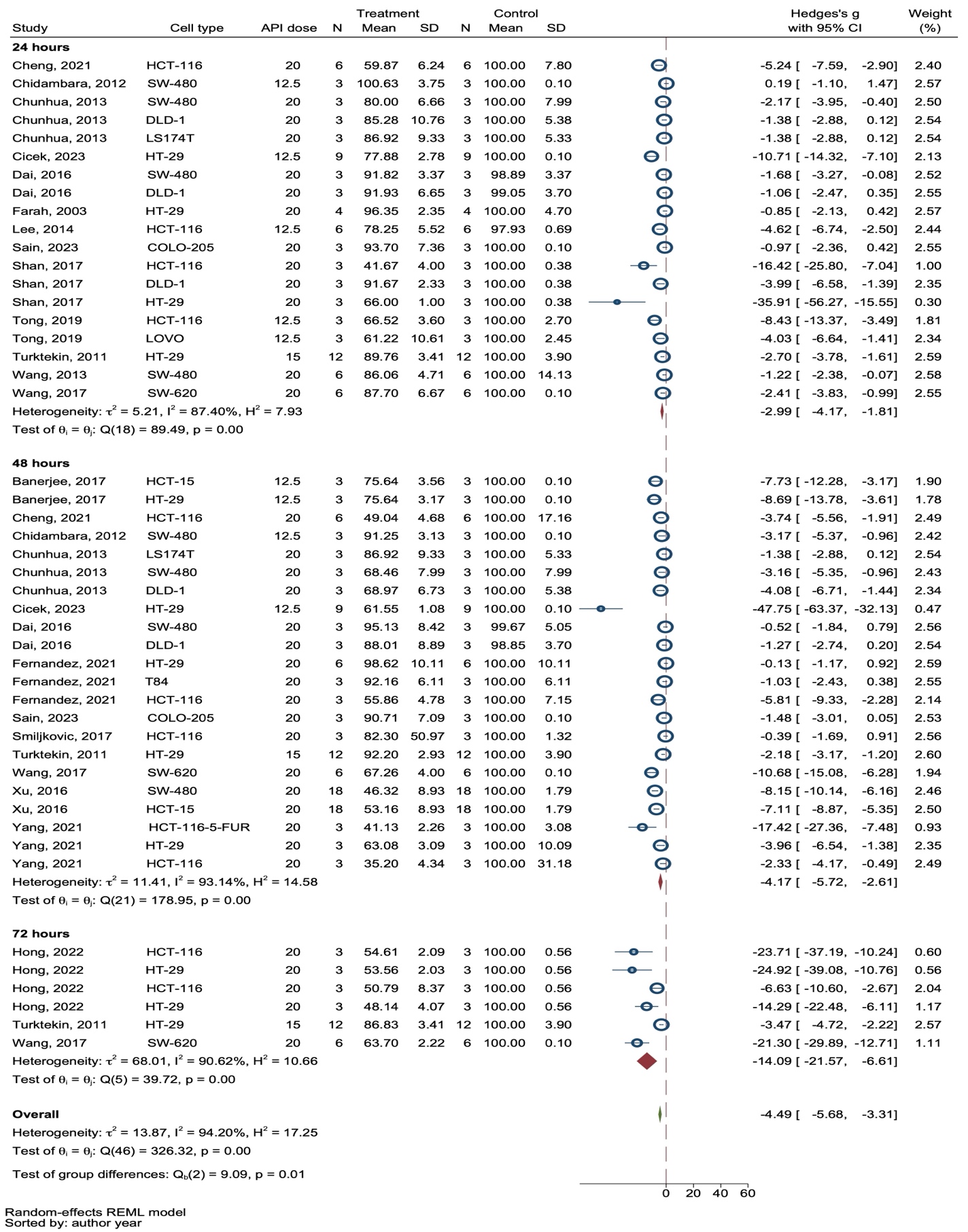
Supplementary Figure 2. Effect of apigenin on cell viability in CRC cell lines with administered dosages between 10.1 and 20 μM.


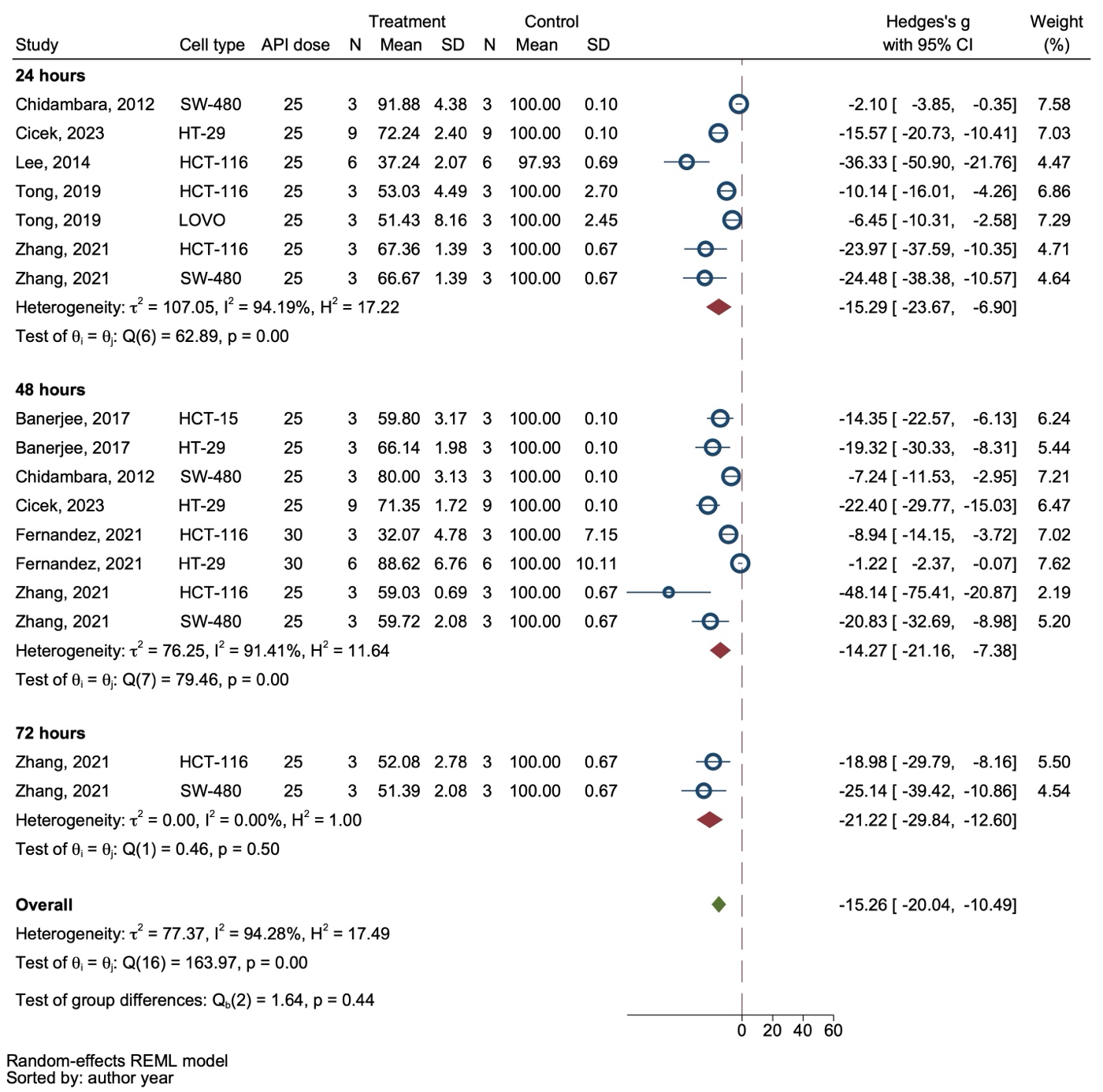


## Supplementary Figure 3. Effect of apigenin on cell viability in CRC cell lines with administered dosages between 20.1 and 30 μM.


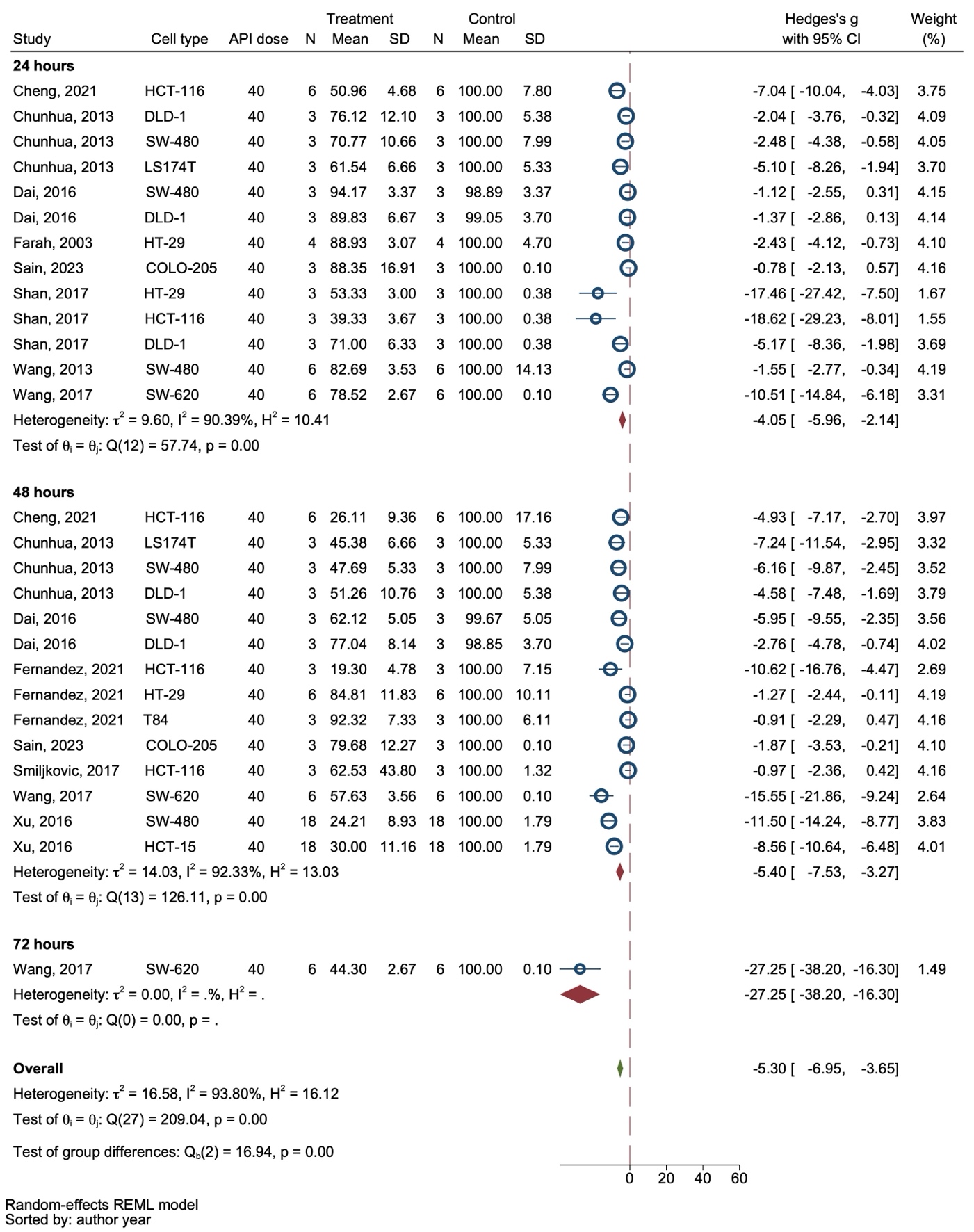


## Supplementary Figure 4. Effect of apigenin on cell viability in CRC cell lines with administered dosages between 30.1 and 40 μM.


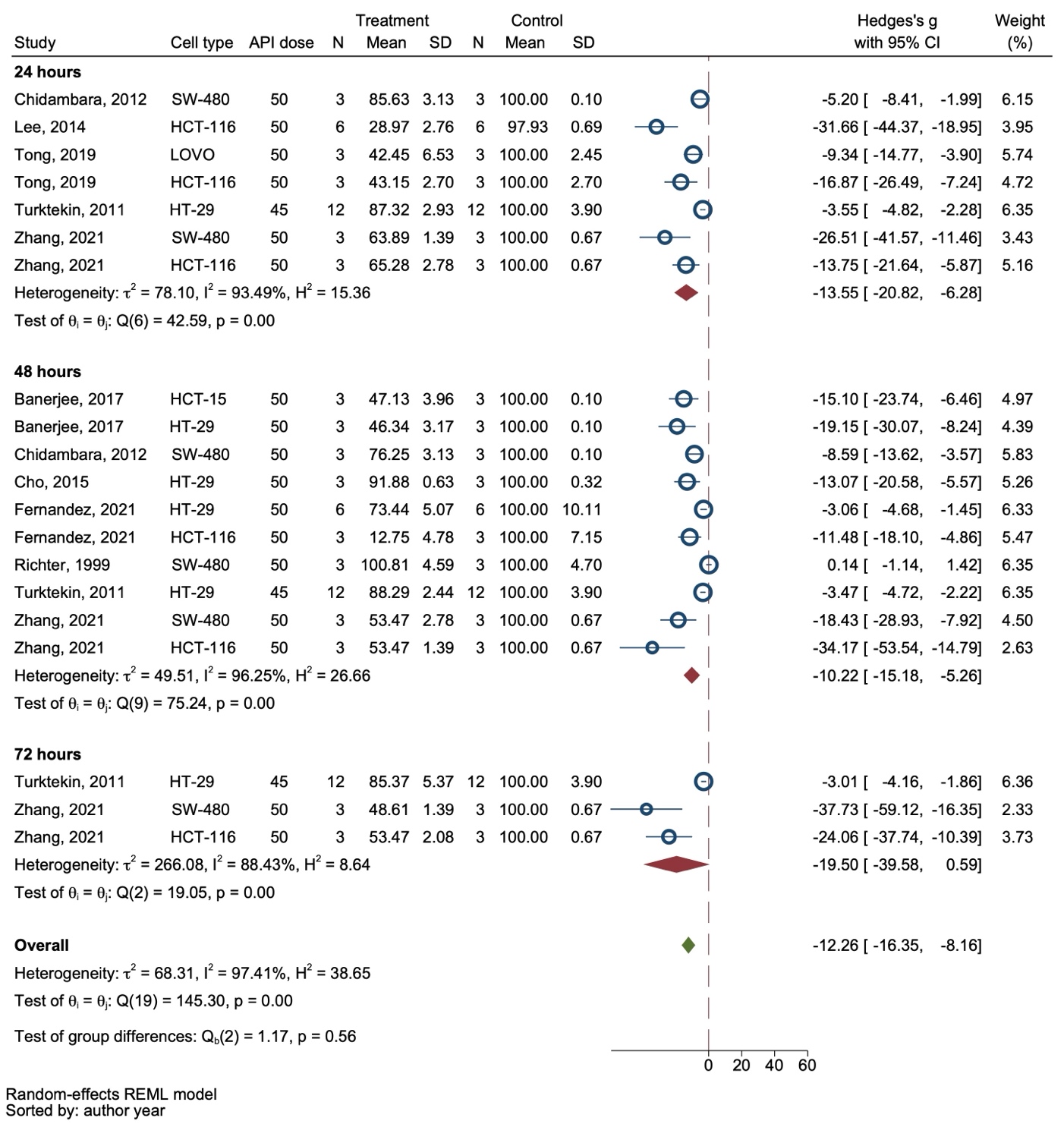


## Supplementary Figure 5. Effect of apigenin on cell viability in CRC cell lines with administered dosages between 40.1 and 50 μM.


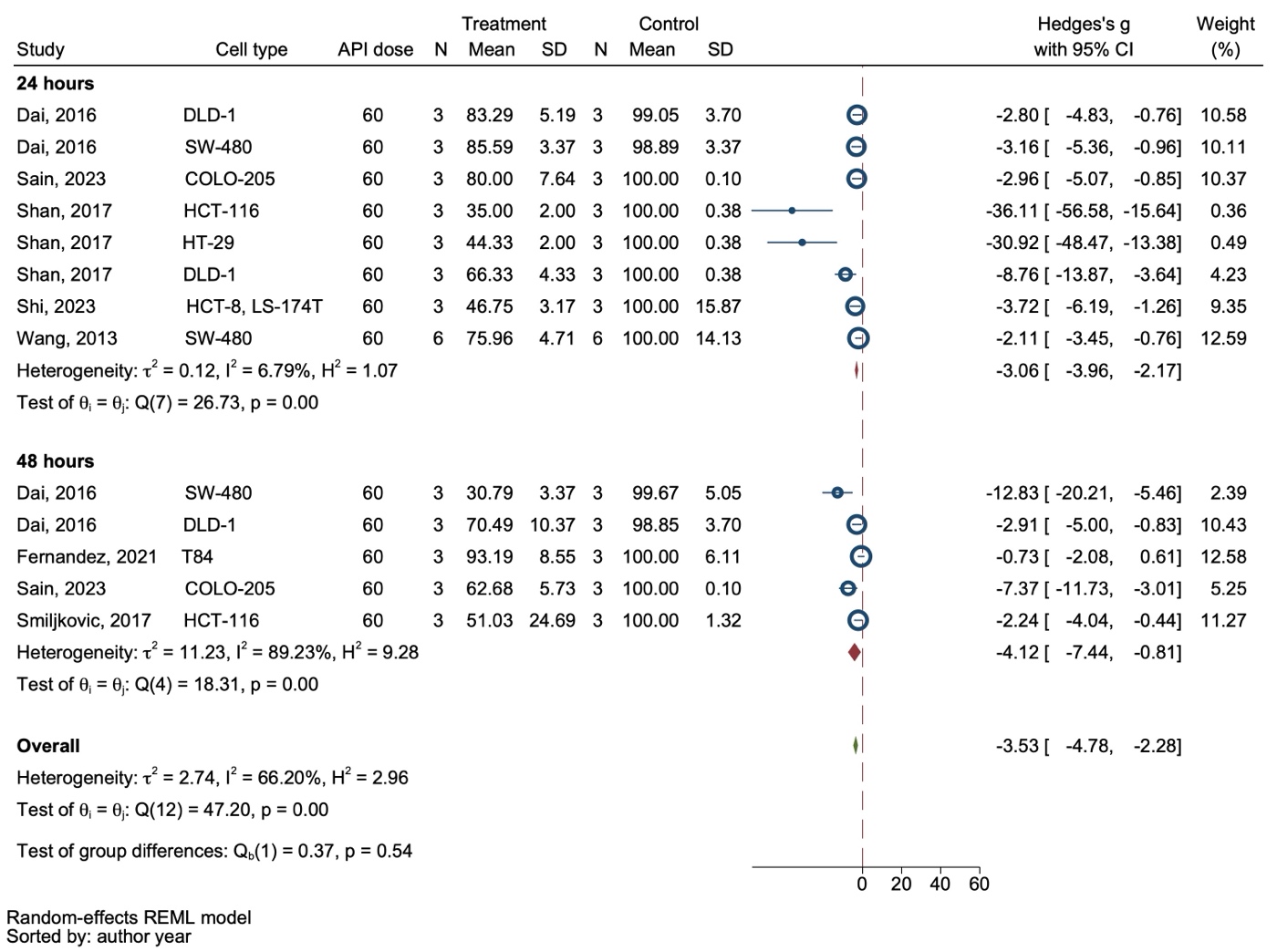


## Supplementary Figure 6. Effect of apigenin on cell viability in CRC cell lines with administered dosages between 50.1 and 60 μM.


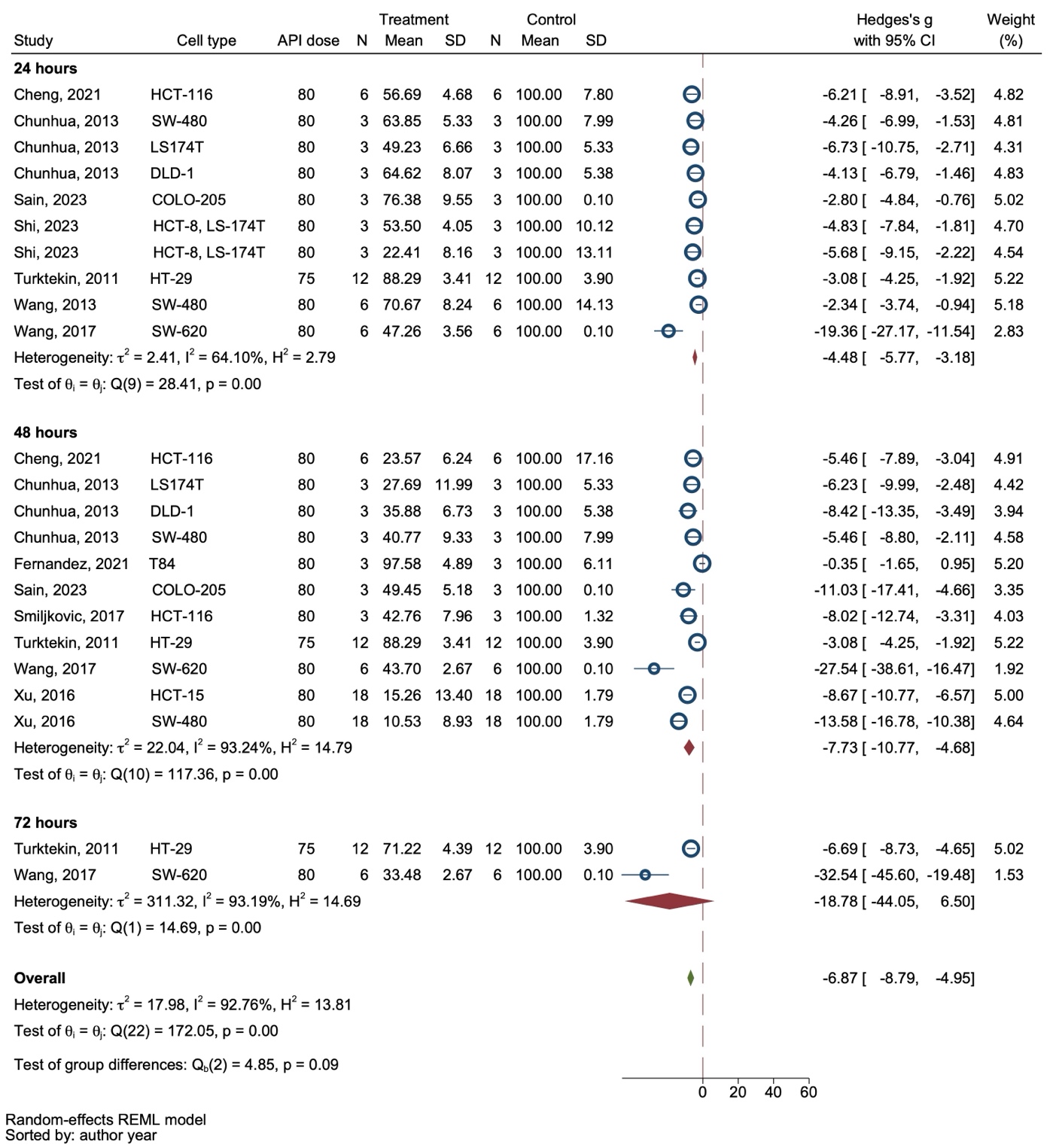


## Supplementary Figure 7. Effect of apigenin on cell viability in CRC cell lines with administered dosages between 70.1 and 80 μM.


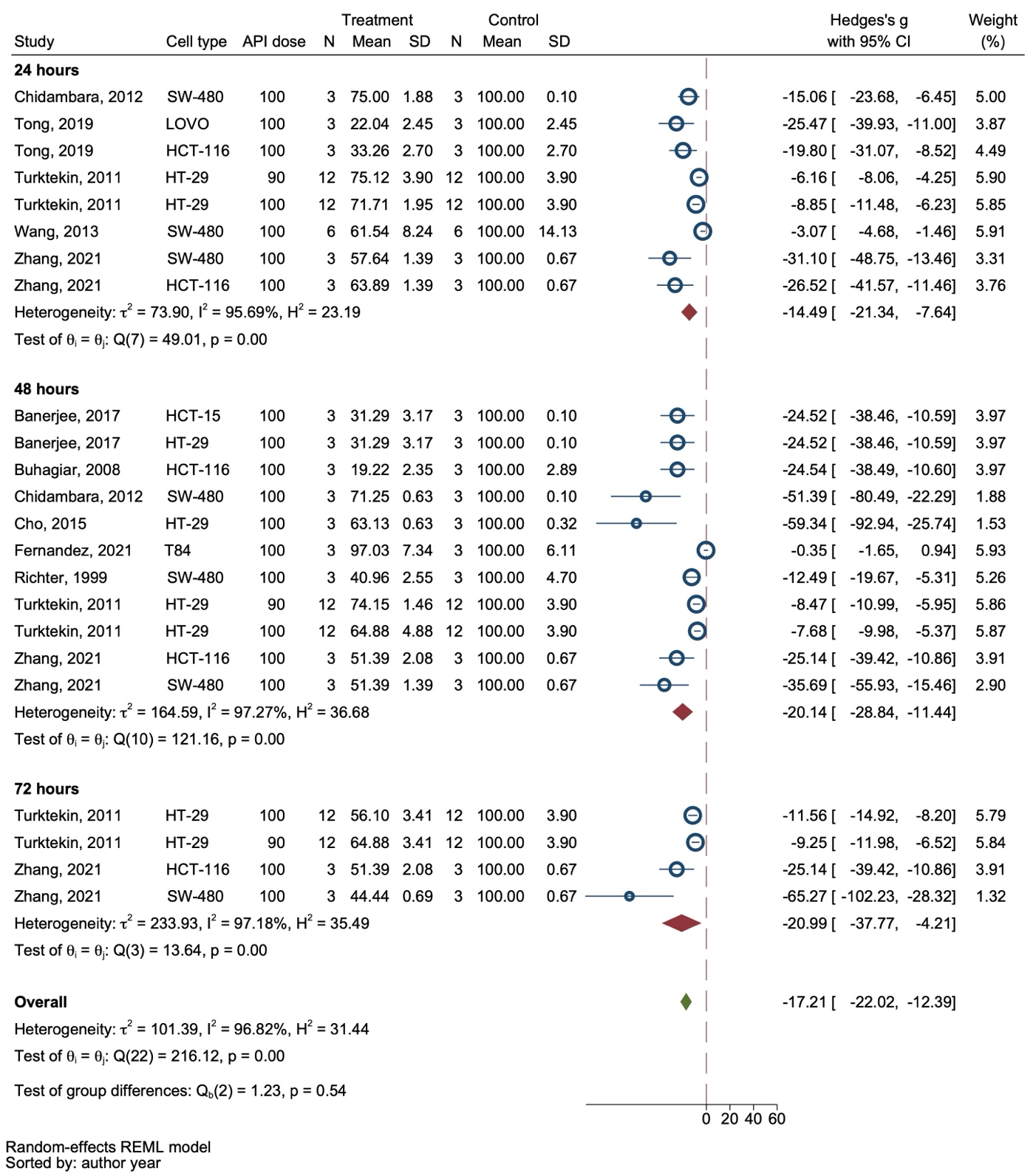


## Supplementary Figure 8. Effect of apigenin on cell viability of CRC cell lines with administered dosages of 90.1 to 100 μM.


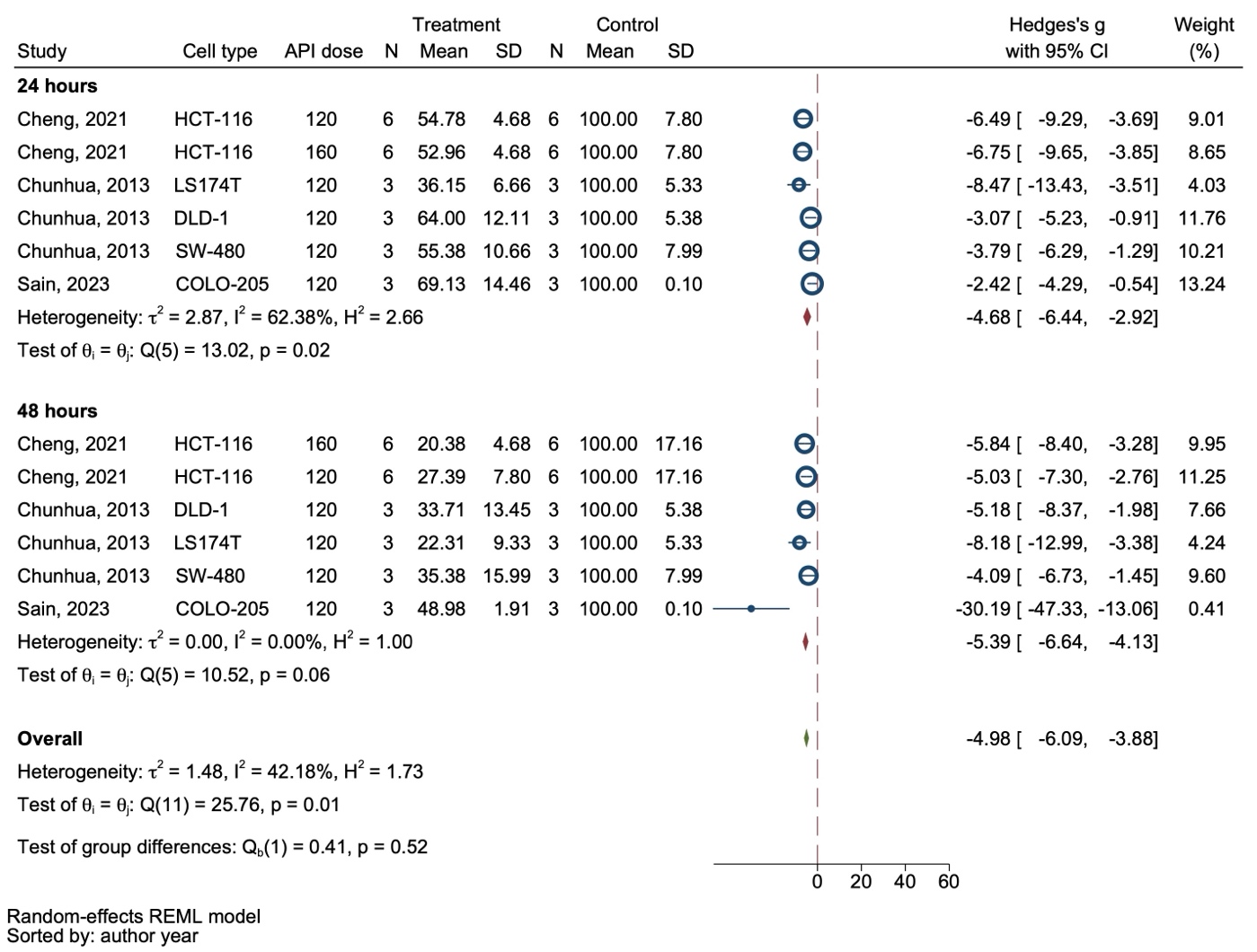


## Supplementary Figure 9. Effect of apigenin on cell viability in CRC cell lines with administered dosages between of 120.1 and 160 μM.


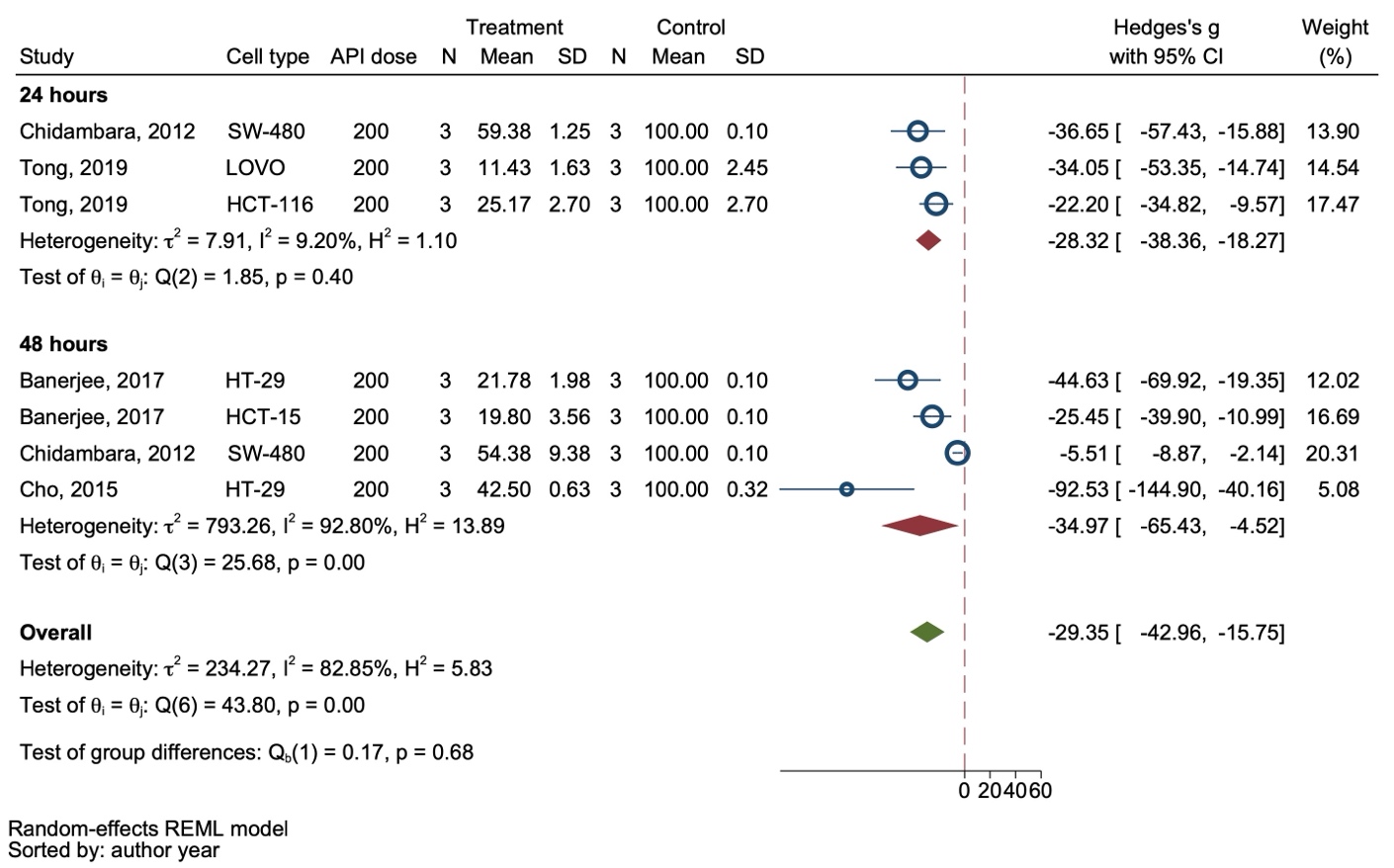


## Supplementary Figure 10. Effect of apigenin on cell viability in CRC cell lines with administered dosages with dosages of 200 μM.


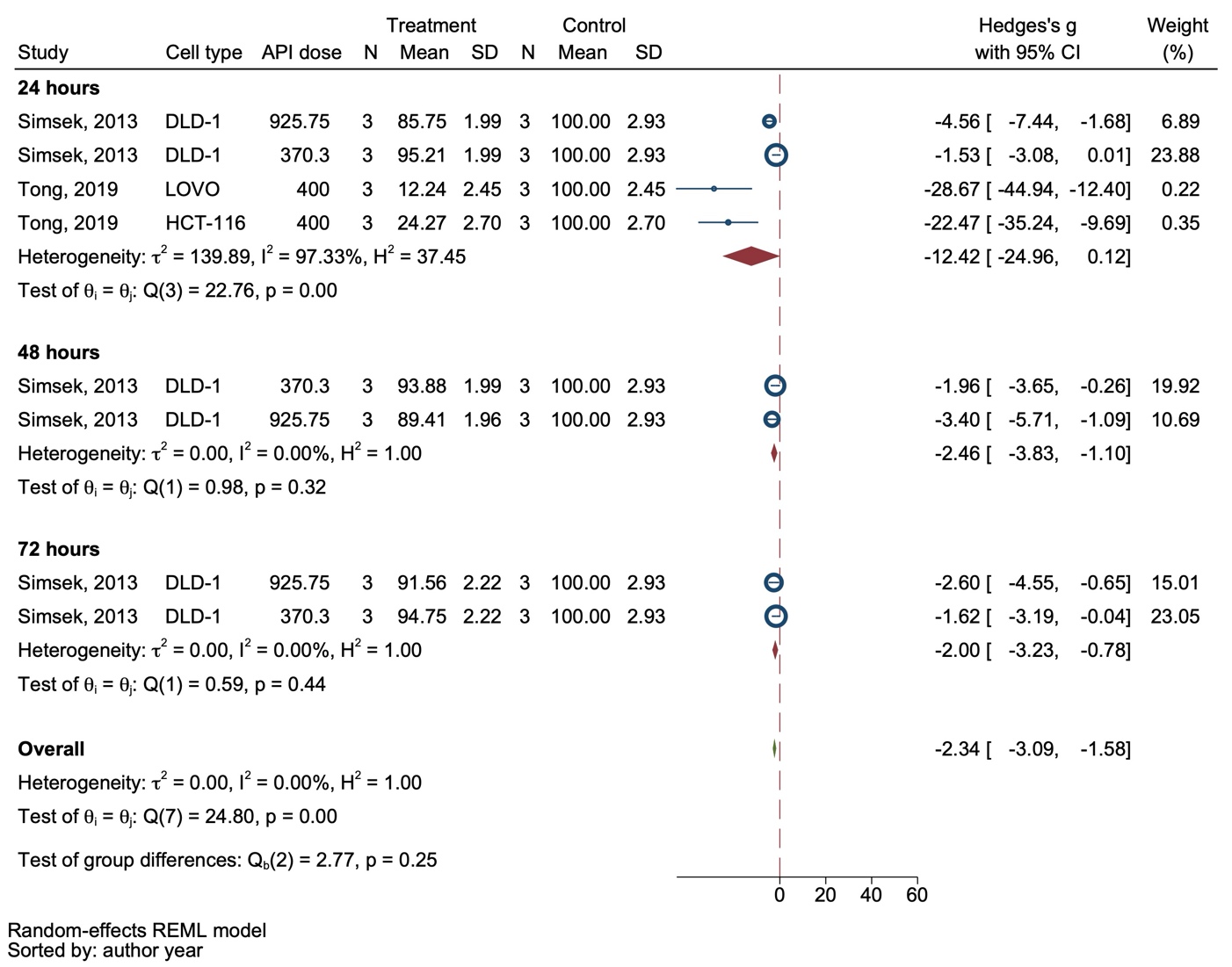


## Supplementary Figure 11. Effect of apigenin on cell viability in CRC cell lines with administered dosages between 200.1 to 1000 μM.


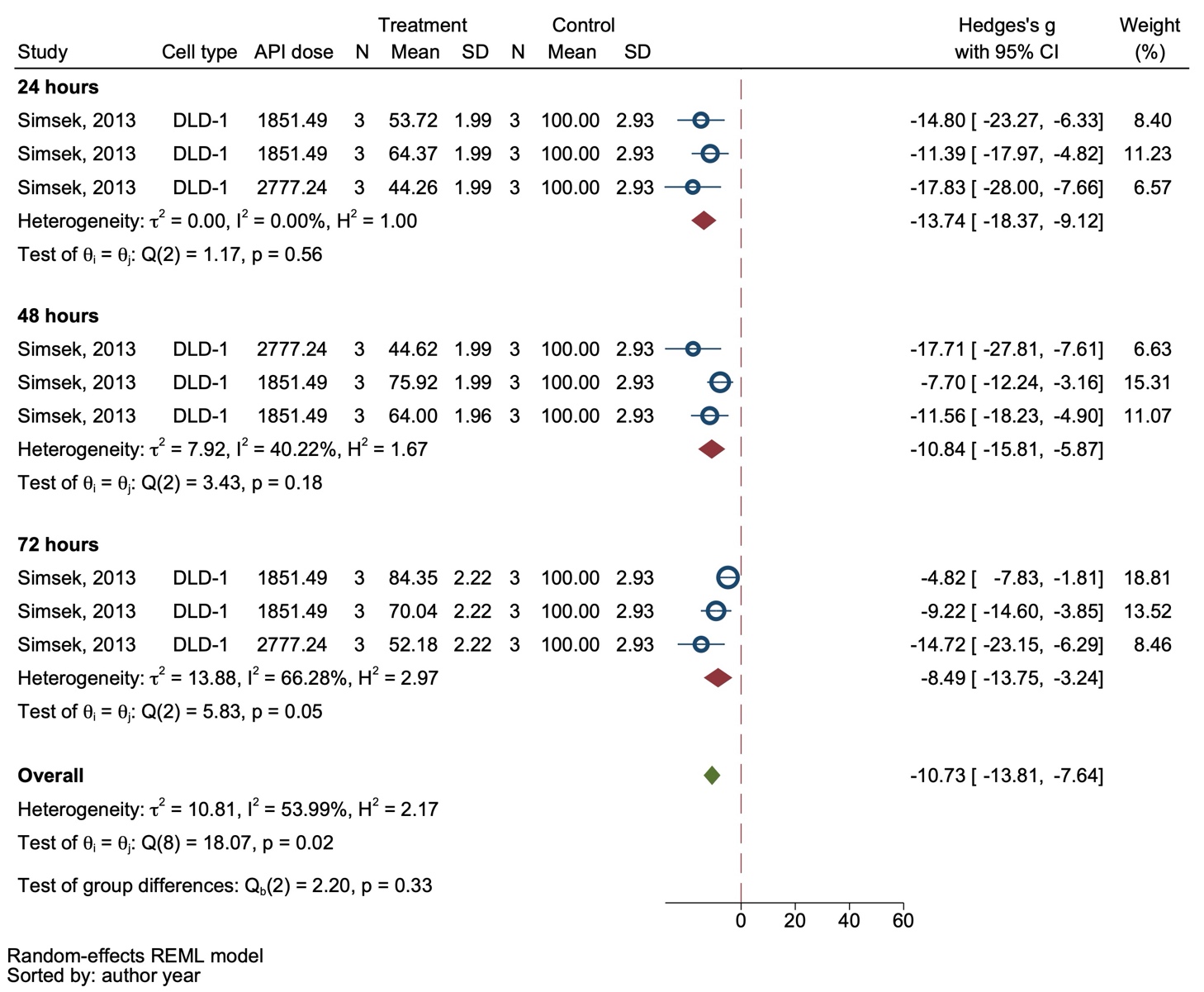


## Supplementary Figure 12. Effect of apigenin on cell viability in CRC cell lines with administered dosages more than 1000.1 μM.

# **Growth Inhibition**

## Supplementary Figure 13. Effect of apigenin on growth inhibition in CRC cell lines with administered dosages ≤ 20 μM.

## Supplementary Figure 14. Effect of apigenin on growth inhibition in CRC cell lines with administered dosages between 20.1 to 40 μM.

## Supplementary Figure 15. Effect of apigenin on growth inhibition with dosages between 60.1 to 80 μM.

## Supplementary Figure 16. Effect of apigenin on growth inhibition with dosages of 120.1 and 160 μM.

# **Apoptosis**

## Supplementary Figure 17. Effect of apigenin on apoptosis with dosages ≤ 10 μM.


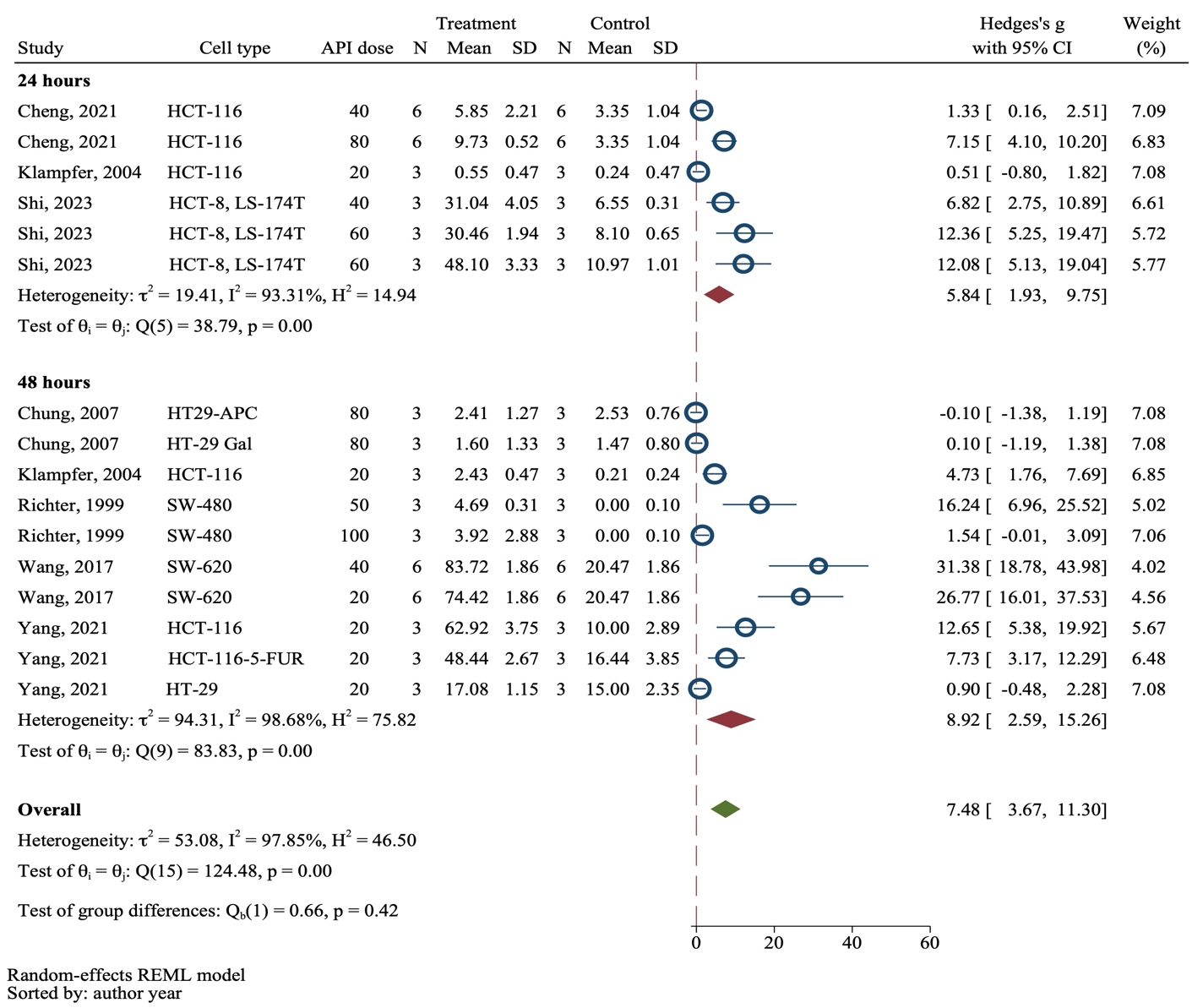


## Supplementary Figure 18. Effect of apigenin on apoptosis with dosages between 10.1 to 100 μM.
